# Altered expression of metabolites and proteins in wild and caged fish exposed to wastewater effluents *in situ*

**DOI:** 10.1038/s41598-017-12473-6

**Published:** 2017-12-05

**Authors:** D. B. D. Simmons, J. Miller, S. Clarence, E. S. McCallum, S. Balshine, B. Chandramouli, J. Cosgrove, J. P. Sherry

**Affiliations:** 1 Aquatic Contaminants Research Division, Water Science and Technology Directorate, Environment and Climate Change Canada, Burlington, ON Canada; 2 Department of Psychology, Neuroscience & Behaviour, McMaster University, Hamilton, ON Canada; 3 Metabolomics Services, SGS AXYS, Sidney, BC Canada

## Abstract

Population growth has led to increased global discharges of wastewater. Contaminants that are not fully removed during wastewater treatment, such as pharmaceuticals and personal care products (PPCPs), may negatively affect aquatic ecosystems. PPCPs can bioaccumulate causing adverse health effects and behavioural changes in exposed fish. To assess the impact of PPCPs on wild fish, and to assess whether caged fish could be used as a surrogate for resident wild fish in future monitoring, we caged goldfish in a marsh affected by discharges of wastewater effluents (Cootes Paradise, Lake Ontario, Canada). We collected plasma from resident wild goldfish, and from goldfish that we caged in the marsh for three weeks. We analyzed the plasma proteome and metabolome of both wild and caged fish. We also compared proteomic and metabolic responses in caged and wild fish from the marsh to fish caged at a reference site (Jordan Harbour Conservation Area). We identified significant changes in expression of over 250 molecules that were related to liver necrosis, accumulation and synthesis of lipids, synthesis of cyclic AMP, and the quantity of intracellular calcium in fish from the wastewater affected marsh. Our results suggest that PPCPs could be affecting the health of wild fish populations.

## Introduction

There is growing societal concern about the environmental fate and inadvertent effects of pharmaceuticals and personal care products (PPCPs). After use or disposal, PPCPs often end-up in wastewater, which then undergoes a multi-step treatment process at municipal wastewater treatment plants (WWTPs) to remove solids, bacteria, and nutrients. WWTPs, however, do not remove all chemical contaminants. In particular, PPCPs have been detected in wastewater effluents and recipient surface waters around the globe^1–4^. Pharmaceuticals are specifically designed to elicit a biological effect in humans. There is growing evidence that these drugs can also have biological effects in non-target organisms that might live in or around recipient waters^5–9^. For example, many PPCPs can also cause endocrine disruption in aquatic organisms^9,10^.

Cootes Paradise Marsh (CPM) is a large and ecologically important wetland on the west side of Hamilton Harbour (ON, Canada). CPM is included in the Hamilton Harbour Area of Concern, which is one of seven Areas of Concern on Lake Ontario identified in the Great Lakes Water Quality Agreement^11^. CPM has suffered considerable habitat destruction and subsequent loss of biodiversity, caused primarily by water pollution from municipal wastewaters (treated effluents and combined sewer overflows), the extensive proliferation of invasive common carp *(Cyprinus carpio)* ^12,13^, and more recently an explosion of goldfish *(Carassius auratus)*. Both carp and goldfish can tolerate a wide range of environmental conditions, including the ability to handle low levels of dissolved oxygen and higher levels of contamination compared to other fish species^14–16^. As part of remediation efforts in CPM, a carp exclusion program was established (and more recently a goldfish removal effort was attempted)^17^, and upgrades were added to the Dundas WWTP. In a previous study of fish captured from CPM, the occurrence of gonadal intersex and elevated plasma vitellogenin was observed in native male white perch (*Morone americana*) captured from CPM. Those reproductive system effects were linked to the potential presence of estrogenic compounds in the marsh water^18^.

**Figure 1:**
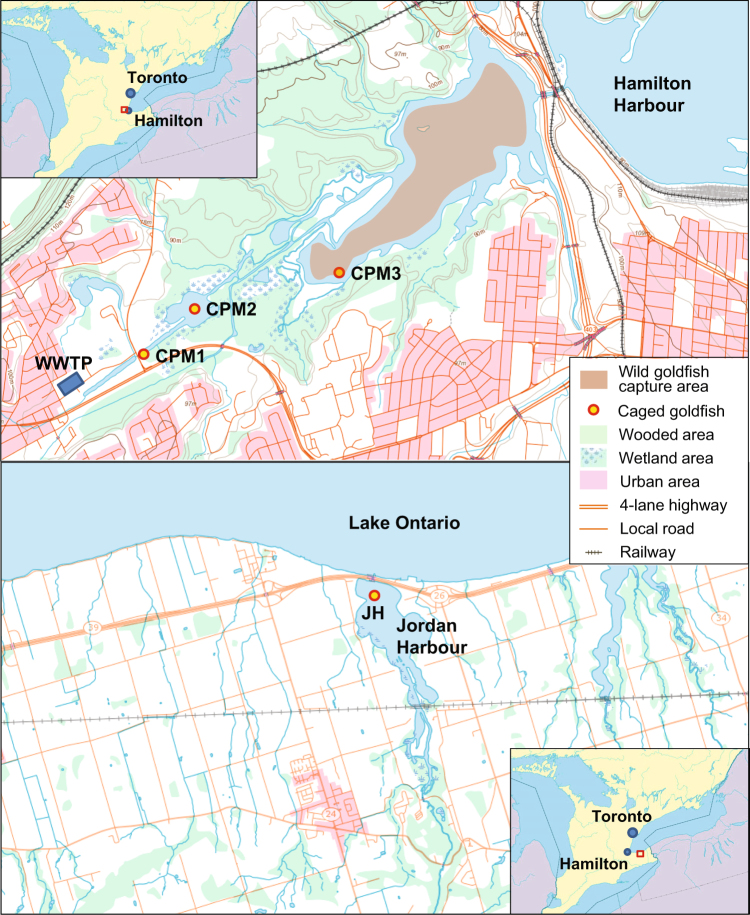
Map of caging and wild fish capture sites in Cootes Paradise Marsh (CPM) and the reference site, Jordan Habour (JH). The base map is from the Atlas of Canada (with permission of Natural Resources Canada).

As part of a larger investigation of the effects of PPCPs in CPM on wild fish^19,20^, the goal of the present study was to investigate if PPCPs present in the treated effluent entering CPM have an impact on wild fish, we collected and tested plasma from both caged fish and wild fish living in CPM for signals of endocrine disruption and molecular level effects. An advantage of blood plasma as a monitoring tool is that it contains molecules from every organ and tissue within the organism as it circulates the entire body. We used responses of the plasma metabolome and proteome to characterize molecular effects. Assuming that a complex mixture of PPCPs would be present in CPM, and that such mixtures could have effects on many different biological functions, we anticipated that our use of multiple ‘omics tools to measure responses in plasma would reveal global molecular responses from the entire organism. We complimented our ‘omics approach with measures of fish survival, plasma vitellogenin, and body morphometrics. We also examined effects in wild goldfish because they are so were abundant in CPM while populations of native fish species are either in decline or in recovery. To link effects observed in wild goldfish specifically to WWTP effluent exposure we caged naïve goldfish as a surrogate for wild fish along a gradient of exposure starting from near the outfall of the Dundas WWTP, and then at two sites further downstream of the outfall and further into CPM (Fig. 1). We also caged goldfish at Jordan Harbour (JH), a conservation area on Lake Ontario and distant from WWTP effluent outfall, as our reference for comparison of responses in both wild-captured and caged goldfish, as we failed to capture wild goldfish in the conservation area (Fig. 1).

## Results

### Survival

There were no acutely lethal effects observed in our caged goldfish – 199 out of 200 fish survived the three-week deployment. The only fish mortality was in a cage at JH and that death was not likely related to exposure or to the caging environment.

### Biometrics

There was no effect of site on the investment in reproductive organs (as measured by the GSI, see methods below) or body condition for the male caged goldfish (Supplementary Table S1).

### Vitellogenin

Vitellogenin (an egg yolk precursor protein, used as a biomarker of environmental estrogen exposure) was detected in the plasma of only one goldfish caged at CPM1 (86 ng/ml) and was also detected in one goldfish caged at JH (27.6 ng/ml).

### Proteins

**Figure 2:**
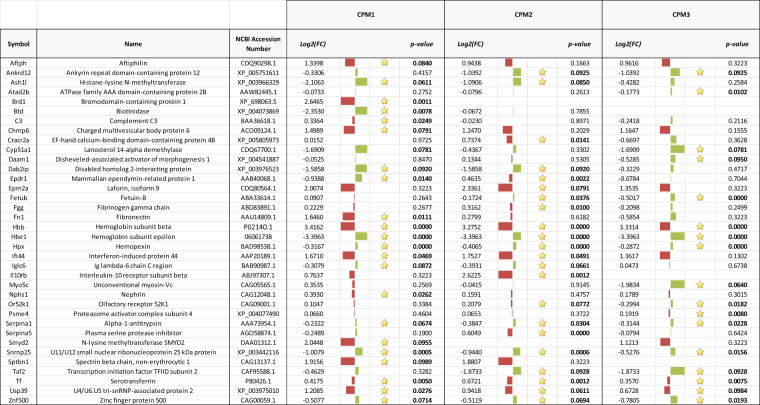
List of proteins with symbol, name, function (if known), fold change (log_2_(FC)), and p-value that were differentially expressed in goldfish plasma for each caging location in CPM compared to expression at the reference site, JH. Red bars indicate increased expression while green bars indicate decreased expression. The size of the bar represents the magnitude of the difference.

**Figure 3:**
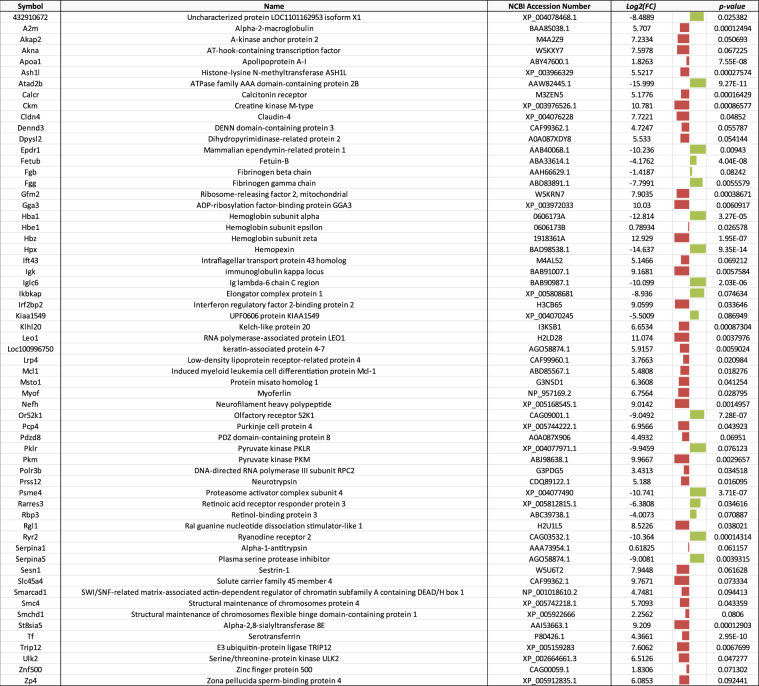
List of proteins with symbol, name, function (if known), fold change (log_2_(FC)), and p-value that were differentially expressed in wild male goldfish plasma from CPM compared to expression in goldfish caged at the reference site, JH. Red bars indicate increased expression while green bars indicate decreased expression. The size of the bar represents the magnitude of the difference.

We employed an untargeted shotgun proteomics approach to identify plasma proteins. Among the plasma proteins we detected in caged male goldfish, the expression of 36 proteins were significantly different in at least one exposure location in CPM compared to the reference site JH (Fig. 2). Of those, 12 were increased and 10 were decreased in goldfish caged closest to the WWTP outfall at CPM1, 11 were increased and 11 were decreased in goldfish caged further away at CPM2, and 4 were increased and 13 were decreased in goldfish caged the farthest from the WWTP outfall at CPM3. In the plasma of wild goldfish captured from CPM, the expression of 43 proteins was significantly increased and 18 proteins were significantly decreased compared to goldfish caged at the reference site JH (Fig. 3). Upon visual inspection of the fold changes values, the expression patterns of 26 proteins demonstrated a trend that could be related to distance along the plume from the WWTP outfall (either CPM1 ≥ CPM2 ≥ CPM3 or CPM3 ≥ CPM2 ≥ CPM1). Protein search scores, percent protein coverage, and accession numbers are included in Supplementary Table S2; further details for single peptide IDs are included in Supplementary Table S3. On average, the log2 fold change for plasma proteins in wild goldfish was 48x greater than for the caged goldfish (48 ± 19; mean (|log2FC_wild_|/|log2FC_caged_|) ± 95% CI). Of the 36 proteins in caged goldfish at CPM that were identified as being significantly different compared to the goldfish caged at the reference JH, 14 were also identified as significantly different in the wild goldfish from CPM. Among those 14 proteins, 6 proteins from fish caged at CPM1, 5 proteins from fish caged at CPM2, and 8 proteins from fish caged at CPM3 were differentially expressed in the same direction as in the wild goldfish plasma.

### Metabolites

**Figure 4:**
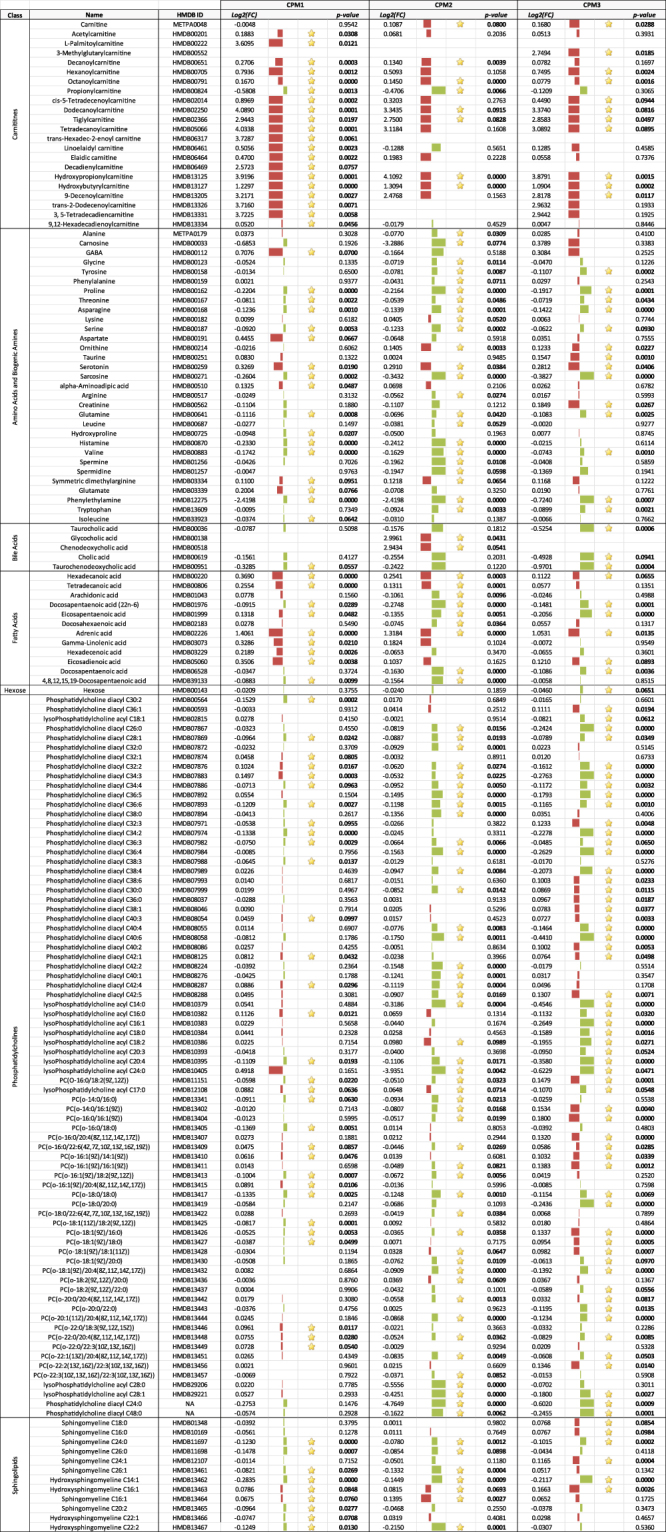
List of metabolites with class of molecule, common name, human metabolome database identifier (HMDB ID), fold change (log_2_(FC)), and p-value that were differentially expressed in goldfish plasma for each caging location in CPM compared to expression in plasma collected from goldfish at the reference site, JH. Red bars indicate increased expression while green bars indicate decreased expression. The size of the bar represents the magnitude of the difference.

**Figure 5:**
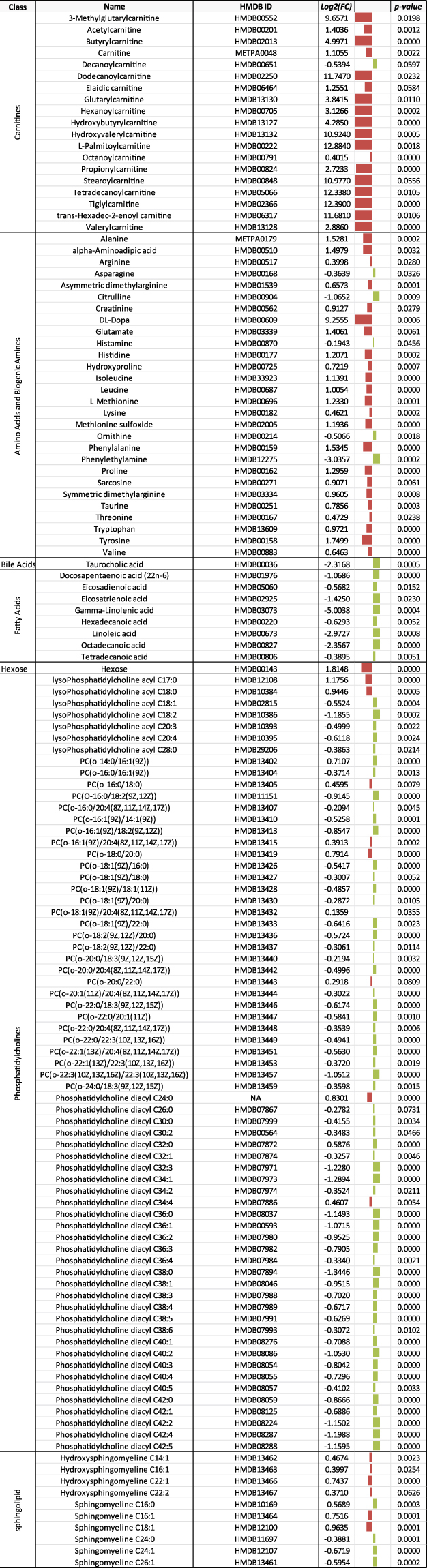
List of metabolites with class of molecule, common name, human metabolome database identifier (HMDB ID), fold change (log_2_(FC)), and p-value that were differentially expressed in plasma from wild male goldfish collected in CPM compared to expression in the plasma of goldfish caged at the reference site, JH. Red bars indicate increased expression while green bars indicate decreased expression. The size of the bar represents the magnitude of the difference.

We used a targeted method to quantify plasma metabolites. Of the 218 targets, we detected 194 metabolites in the plasma samples from the caged goldfish (Fig. 4). Of those, the concentrations of 48 increased and 39 decreased in goldfish caged closest to the WWTP outfall at CPM1 compared to fish at the reference site JH. In goldfish caged further away at CPM2, 22 metabolites increased and 74 decreased. Finally, 44 metabolites increased and 54 decreased in goldfish farthest from the WWTP outfall at CPM3. For the wild goldfish from CPM, 27 of the 218 metabolite targets were not detected in the plasma of any fish. Of those metabolites that were detected, the concentrations of 77 were decreased and 57 were increased compared to the plasma of fish caged at JH (Fig. 5). Upon visual inspection of the fold changes values, 58 metabolites demonstrated expression patterns that could be related to distance from the WWTP outfall (either CPM1 ≥ CPM2 ≥ CPM3 or CPM > CPM2 ≥ CPM1). On average, the log2 fold change for plasma metabolites in wild goldfish was 31x greater than for the caged goldfish (31 ± 10; mean (|log2FC_wild_|/|log2FC_caged_|) ± 95% CI). Of the 159 metabolites in caged goldfish at CPM that were identified as being significantly different compared to the goldfish caged at the reference JH, 109 were also identified as significantly different in the wild goldfish from CPM. Among those 109 metabolites, 58 metabolites from fish caged at CPM1, 65 metabolites from fish caged at CPM2, and 51 metabolites from fish caged at CPM3 were differentially expressed in the same direction as in the wild goldfish plasma.

### Biological functions

The Ingenuity Pathways Analysis (IPA core analysis) identified 47 biological functions that were considered significantly activated or inhibited, based upon expression of both plasma proteins and metabolites of caged male goldfish and wild male goldfish from CPM compared to fish from the reference site at JH (Table 1). In fish caged closest to the outflow at CPM1, liver necrosis functions, and metal ion transport were activated, and synthesis of cyclic AMP was inhibited. Uptake of amino acids was activated, while growth of organism and entry into S-phase cell division were inhibited in fish caged further from the WWTP outfall at CPM2. At CPM3, furthest from the WWTP outfall, accumulation of lipids and glyceride were activated, and growth of organism, synthesis of cyclic AMP, and quantity of steroid were inhibited. In the wild male goldfish from CPM, cell survival, concentration of glutathione, and contractility of heart were activated, whereas apoptosis was inhibited. Additionally, IPA analysis identified 6 similar functions that were affected in the wild and caged goldfish (uptake of amino acids, uptake of L-amino acid, uptake of L-alanine, quantity of metal, quantity of Ca^2+^, and accumulation of lipids), but their predicted activation states were in opposite directions (Table 1).

**Table 1:** List of biological functions with activation scores (z-value), p-value, and list of the molecules (either the gene symbol ortholog for proteins or the common name for metabolites) identified as being related to that function.

		Biological Function	Activation z-score	p-alue	# Mols	Molecules
Caged Male Goldfish	CPM1	synthesis of cyclic AMP	−2.501	1.48E-04	7	2-phenethylamine,5-hydroxytryptamine,cholic acid,histamine,palmitic acid,taurochenodeoxycholate,taurocholic acid
		biosynthesis of cyclic nucleotides	−2.028	4.35E-05	8	2-phenethylamine,5-hydroxytryptamine,cholic acid,histamine,myristic acid,palmitic acid,taurochenodeoxycholate,taurocholic acid
		efflux of neutral amino acid	−2	1.04E-07	4	glycine,L-leucine,L-serine,L-threonine
		efflux of L-amino acid	−2	2.46E-06	4	glycine,L-leucine,L-serine,L-threonine
		cell death of liver cells	2.374	5.53E-06	8	arachidonic acid,cholic acid,glycine,histamine,L-arginine,palmitic acid,SPTBN1,taurine
		necrosis of liver	2.326	2.55E-07	10	arachidonic acid,C3,cholic acid,glycine,histamine,L-arginine,L-phenylalanine,palmitic acid,SPTBN1,taurine
		quantity of Ca2+	2.253	8.60E-13	20	(all Z)-7,10,13,16,19-docosapentaenoic acid,5-hydroxytryptamine,9Z-hexadecenoic acid,adrenic acid,arachidonic acid,C3,CRACR2A,docosahexaenoic acid,eicosa-11Z, 14Z-dienoic acid,FN1,GABA,gamma-linolenic acid,glycine,histamine,icosapent,L-lysine,L-ornithine,myristic acid,NPHS1,palmitic acid
		cell death of hepatocytes	2.185	1.00E-05	7	arachidonic acid,cholic acid,glycine,histamine,L-arginine,palmitic acid,SPTBN1
		quantity of metal	2.091	2.78E-13	22	(all Z)-7,10,13,16,19-docosapentaenoic acid,5-hydroxytryptamine,9Z-hexadecenoic acid,adrenic acid,arachidonic acid,C3,CRACR2A,docosahexaenoic acid,eicosa-11Z, 14Z-dienoic acid,FN1,GABA,gamma-linolenic acid,glycine,histamine,HPX,icosapent,L-lysine,L-ornithine,myristic acid,NPHS1,palmitic acid,TF
		apoptosis of liver cells	2.023	9.15E-06	7	arachidonic acid,glycine,histamine,L-arginine,palmitic acid,SPTBN1,taurine
	CPM2	growth of bacteria	−2.739	8.53E-12	12	docosahexaenoic acid,glycine,L-arginine,L-aspartic acid,L-phenylalanine,L-proline,L-serine,L-threonine,L-valine,spermidine,spermine,TF
		entry into S phase of hepatocytes	−2.236	2.30E-11	5	glycine,L-asparagine,L-aspartic acid,L-proline,L-serine
		entry into S phase	−2.236	2.52E-05	6	FN1,glycine,L-asparagine,L-aspartic acid,L-proline,L-serine
		export of molecule	−2.047	2.31E-07	12	arachidonic acid,cholic acid,docosahexaenoic acid,GABA,glycine,L-aspartic acid,L-leucine,L-serine,L-threonine,spermine,sphingomyelin,taurocholic acid
		growth of organism	−2.043	2.78E-04	14	docosahexaenoic acid,FN1,GABA,glycine,L-arginine,L-aspartic acid,L-phenylalanine,L-proline,L-serine,L-threonine,L-valine,spermidine,spermine,TF
		excitation of orexin neurons	−2	6.17E-08	4	glycine,L-aspartic acid,L-proline,L-serine
		efflux of neutral amino acid	−2	8.89E-08	4	glycine,L-leucine,L-serine,L-threonine
		efflux of L-amino acid	−2	2.10E-06	4	glycine,L-leucine,L-serine,L-threonine
		uptake of amino acids	3.062	9.63E-16	14	D-tryptophan,GABA,glycine,isoleucine,L-aspartic acid,L-phenylalanine,L-serine,L-threonine,L-tyrosine,sarcosine,spermine,taurine,taurocholic acid,trans-4-hydroxy-L-proline
		uptake of L-amino acid	2.897	1.76E-16	13	D-tryptophan,GABA,glycine,isoleucine,L-aspartic acid,L-phenylalanine,L-serine,L-threonine,L-tyrosine,sarcosine,spermine,taurine,trans-4-hydroxy-L-proline
		uptake of L-alanine	2.449	4.94E-11	6	glycine,isoleucine,L-phenylalanine,L-serine,L-threonine,L-tyrosine
		blood pressure	2.138	1.03E-04	8	5-hydroxytryptamine,arachidonic acid,GABA,glycine,histamine,L-arginine,L-lysine,L-ornithine
Caged Male Goldfish	CPM2	uptake of glutamine family amino acid	2.121	1.64E-10	9	D-tryptophan,GABA,glycine,L-aspartic acid,L-phenylalanine,sarcosine,spermine,taurine,trans-4-hydroxy-L-proline
		transport of amino acids	2.053	1.12E-15	16	D-tryptophan,GABA,glycine,isoleucine,L-aspartic acid,L-leucine,L-lysine,L-phenylalanine,L-serine,L-threonine,L-tyrosine,sarcosine,spermine,taurine,taurocholic acid,trans-4-hydroxy-L-proline
	CPM3	growth of organism	−2.554	2.11E-04	14	docosahexaenoic acid,FN1,GABA,glycine,L-arginine,L-aspartic acid,L-phenylalanine,L-proline,L-serine,L-threonine,L-valine,spermidine,spermine,TF
		synthesis of cyclic AMP	−2.501	9.78E-05	7	2-phenethylamine,5-hydroxytryptamine,cholic acid,histamine,palmitic acid,taurochenodeoxycholate,taurocholic acid
		quantity of steroid	−2.306	8.22E-05	11	5-hydroxytryptamine,acetyl-L-carnitine,arachidonic acid,carnosine,cholic acid,docosahexaenoic acid,EPM2A,histamine,palmitic acid,sphingomyelin,taurine
		transport of molecule	−2.279	1.58E-11	33	2-phenethylamine,5-hydroxytryptamine,arachidonic acid,C3,cholic acid,creatinine,D-tryptophan,docosahexaenoic acid,FGG,FN1,GABA,glycine,HBB,histamine,HPX,isoleucine,L-aspartic acid,L-leucine,L-lysine,L-phenylalanine,L-serine,L-threonine,L-tyrosine,myristic acid,palmitic acid,sarcosine,spermine,sphingomyelin,taurine,taurochenodeoxycholate,taurocholic acid,TF,trans-4-hydroxy-L-proline
		release of acidic amino acid	−2.219	2.25E-06	6	2-aminoadipic acid,5-hydroxytryptamine,GABA,glycine,histamine,L-arginine
		release of L-amino acid	−2.219	2.62E-06	6	2-aminoadipic acid,5-hydroxytryptamine,GABA,glycine,histamine,L-arginine
		Fibrosis	−2.219	3.42E-03	9	C3,cholic acid,docosahexaenoic acid,FN1,GABA,HBB,HPX,L-arginine,taurine
		growth of bacteria	−2.191	6.30E-12	12	docosahexaenoic acid,glycine,L-arginine,L-aspartic acid,L-phenylalanine,L-proline,L-serine,L-threonine,L-valine,spermidine,spermine,TF
		exocytosis	−2.162	1.32E-03	5	arachidonic acid,FGG,glycine,histamine,spermine
		proliferation of cells	−2.073	3.12E-03	31	2-phenethylamine,5-hydroxytryptamine,arachidonic acid,C3,cholic acid,DAB2IP,docosahexaenoic acid,FN1,GABA,gamma-linolenic acid,glycine,histamine,HPX,L-arginine,L-asparagine,L-aspartic acid,L-lysine,L-phenylalanine,L-proline,L-serine,L-threonine,L-valine,myristic acid,palmitic acid,sarcosine,SERPINA1,SERPINA5,spermidine,spermine,taurocholic acid,TF
		biosynthesis of cyclic nucleotides	−2.028	2.70E-05	8	2-phenethylamine,5-hydroxytryptamine,cholic acid,histamine,myristic acid,palmitic acid,taurochenodeoxycholate,taurocholic acid
		efflux of neutral amino acid	−2	7.98E-08	4	glycine,L-leucine,L-serine,L-threonine
		efflux of L-amino acid	−2	1.89E-06	4	glycine,L-leucine,L-serine,L-threonine
		accumulation of acylglycerol	2.162	6.93E-05	5	arachidonic acid,cholic acid,L-arginine,myristic acid,palmitic acid
		accumulation of lipid	2.044	2.18E-06	11	5-hydroxytryptamine,arachidonic acid,cholic acid,docosahexaenoic acid,FN1,L-arginine,L-serine,myristic acid,palmitic acid,sphingomyelin,taurine
CPM Wild Male Goldfish		uptake of L-amino acid	−2.985	1.17E-10	10	D-tryptophan,isoleucine,L-histidine,L-methionine,L-phenylalanine,L-threonine,L-tyrosine,sarcosine,taurine,trans-4-hydroxy-L-proline
		apoptosis	−2.86	3.48E-03	28	A2M,acetyl-L-carnitine,APOA1,CALCR,HBA1/HBA2,histamine,IKBKAP,IRF2BP2,KLHL20,L-arginine,L-histidine,L-methionine,L-phenylalanine,L-tyrosine,linoleic acid,MCL1,myristic acid,N,N-dimethylarginine,palmitic acid,PCP4,PKM,RBP3,RYR2,SERPINA1,stearic acid,taurine,TF,ULK2
		cell death	−2.45	3.06E-03	34	2-aminoadipic acid,A2M,acetyl-L-carnitine,APOA1,CALCR,CLDN4,colfosceril palmitate,FETUB,HBA1/HBA2,histamine,IKBKAP,IRF2BP2,KLHL20,L-arginine,L-histidine,L-methionine,L-phenylalanine,L-tyrosine,linoleic acid,MCL1,myristic acid,N,N-dimethylarginine,NEFH,palmitic acid,PCP4,PKLR,PKM,RBP3,RYR2,SERPINA1,stearic acid,taurine,TF,ULK2
		uptake of L-alanine	−2.449	1.71E-10	6	isoleucine,L-histidine,L-methionine,L-phenylalanine,L-threonine,L-tyrosine
		uptake of amino acids	−2.373	2.62E-10	11	D-tryptophan,isoleucine,L-histidine,L-methionine,L-phenylalanine,L-threonine,L-tyrosine,sarcosine,taurine,taurocholic acid,trans-4-hydroxy-L-proline
		organismal death	−2.346	4.79E-03	25	2-phenethylamine,A2M,APOA1,CALCR,CLDN4,creatinine,FGG,gamma-linolenic acid,HBZ,histamine,IKBKAP,L-arginine,L-phenylalanine,linoleic acid,LRP4,MCL1,myristic acid,palmitic acid,PKM,RYR2,SERPINA1,stearic acid,TF,TRIP12,ULK2
		quantity of Ca2+	−2.202	2.90E-06	13	A2M,CALCR,gamma-linolenic acid,HBA1/HBA2,histamine,L-lysine,L-ornithine,linoleic acid,MCL1,myristic acid,palmitic acid,RYR2,stearic acid
		quantity of metal	−2.148	8.98E-07	15	A2M,CALCR,gamma-linolenic acid,HBA1/HBA2,histamine,HPX,L-lysine,L-ornithine,linoleic acid,MCL1,myristic acid,palmitic acid,RYR2,stearic acid,TF
CPM Wild Male Goldfish		synthesis of lipid	−2.092	2.15E-03	12	APOA1,histamine,L-arginine,L-methionine,linoleic acid,myristic acid,palmitic acid,PKM,SERPINA1,ST8SIA5,stearic acid,taurocholic acid
		accumulation of lipid	−2.037	2.69E-03	7	APOA1,L-arginine,linoleic acid,myristic acid,palmitic acid,stearic acid,taurine
		uptake of L-proline	−2	8.48E-07	5	D-tryptophan,L-phenylalanine,sarcosine,taurine,trans-4-hydroxy-L-proline
		proliferation of CD4+ T-lymphocytes	−2	1.71E-04	4	gamma-linolenic acid,linoleic acid,palmitic acid,stearic acid
		cell survival	2.872	3.46E-04	20	CALCR,CLDN4,gamma-linolenic acid,HBA1/HBA2,HBZ,histamine,isoleucine,L-arginine,L-histidine,L-methionine,L-proline,LEO1,MCL1,NEFH,palmitic acid,PKLR,PKM,RARRES3,RYR2,stearic acid
		cell viability	2.733	3.77E-04	19	CALCR,CLDN4,gamma-linolenic acid,HBA1/HBA2,HBZ,histamine,isoleucine,L-arginine,L-histidine,L-methionine,L-proline,MCL1,NEFH,palmitic acid,PKLR,PKM,RARRES3,RYR2,stearic acid
		incorporation of thymidine	2.438	8.14E-06	6	L-methionine,linoleic acid,myristic acid,palmitic acid,stearic acid,TF
		concentration of glutathione	2.042	1.02E-06	7	acetyl-L-carnitine,citrulline,gamma-linolenic acid,L-arginine,L-methionine,PKM,taurine
		oxidation of glucose-6-phosphate	2	2.00E-08	4	linoleic acid,myristic acid,palmitic acid,stearic acid
		contractility of heart	2	1.96E-03	5	APOA1,CKM,L-arginine,MCL1,RYR2

## Discussion

**Figure 6:**
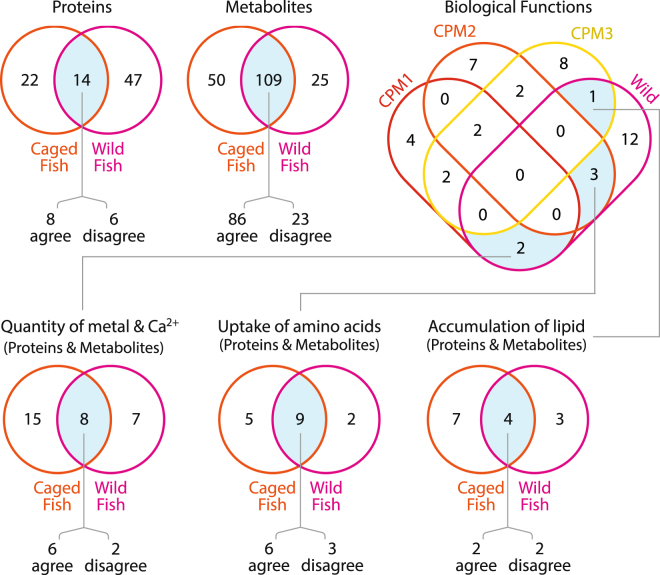
Venn diagrams of either the number of molecules (proteins and metabolites) or the number of biological functions in caged and wild goldfish. Intersections contain the number of molecules or functions that were common to both caged and wild goldfish (highlighted in blue). The word “agree” indicates the number of molecules where the direction of the fold change was the same for both caged and wild goldfish, and the word “disagree” indicates the number of molecules whose expression was in the opposite direction for the caged and wild goldfish. Where there was more than one biological function in an intersection, the number of molecules related to those functions were pooled together to make one Venn diagram for the multiple funcions ﻿(quantity of metal and quantity of Ca2+ merged to quantity of metal and Ca2+, and uptake of L-amino acids, uptake of amino acids, and uptake of L-alanine were merged to uptake of amino acids).

We used an ‘omics approach to understand the molecular effects of exposure to wastewater effluent in goldfish caged for three weeks compared to wild fish that would have been chronically exposed throughout their lifetime. For proteins and metabolites together, there was close to 75% agreement in the direction of fold change expression for caged and wild goldfish plasma at wastewater-exposed CPM sites compared to the reference JH. The targeted metabolomics data better predicted responses in wild goldfish than did the untargeted protein data (79% versus 57%, Fig. 6), which is likely explained by the higher variation that accompanies untargeted approaches^21^. The advantage of untargeted approaches are that they are less biased, however they come with the cost of lowered precision - and while targeted approaches are more precise, they do not capture as much information as untargeted methods. By employing both strategies in the present study (untargeted proteomics and targeted metabolomics), we strived to achieve a balanced design. The magnitude of protein and metabolite expression fold change was greater in wild goldfish compared to the caged goldfish, possibly reflecting that the caged goldfish were exposed for only 21 days in CPM, while the wild goldfish presumably spent much of their lifetime in the marsh and had more time to adjust to their environment. Differences among the three sites were more apparent after functional biological analyses; only 6 out of 43 of the significantly affected biological functions were common to the wild and caged fish. Furthermore, among those 6 common biological functions, all were considered to be significantly activated in the caged fish, but inhibited in wild goldfish on the basis of the IPA derived z-scores.

In the present study, we detected 10 molecules in the plasma of goldfish caged nearest the outflow of the WWTP at CPM1 that were identified by the IPA core analysis as being involved in the activation of liver and liver cell necrosis (Table 1). We also detected 15 PPCPs (out of 127 targets) in the plasma of goldfish caged nearest to the WWTP outfall CPM1^19,20^, among which, 7 were psychotropic drugs or their metabolites (amitriptyline, citalopram, fluoxetine/norfluoxetine, sertraline, venlafaxine, and oxazepam) and three were antimicrobials (erythromycin-H_2_O, flumequine, and sulfamethazine)(plasma concentrations of PPCPs are summarized in Table 2). Naproxen and ibuprofen (both non-steroidal anti-inflammatory drugs; NSAIDs) were detected in the water of CPM, but were below detection limits in the plasma. Additionally, the antidepressant drug fluoxetine and the fibrate drug gemfibrozil had the highest bioaccumulation factors (BAFs) in caged and wild goldfish^20^.

**Table 2:** Blood plasma concentrations of PPCPs in pooled plasma from caged goldfish and individual plasma samples from wild goldfish (adapted from Muir *et al*.^20^).

PPCP name	Use	JH caged goldfish (n_pooled_ = 1)	CPM1 caged goldfish (n_pooled_ = 1)	CPM2 caged goldfish (n_pooled_ = 1)	CPM3 caged goldfish (n_pooled_ = 1)	CPM wild goldfish n = 3
		ng/g	ng/g	ng/g	ng/g	Mean (range) ng/g
Hydrocortisone*	Steroidal Anti-inflammatory	92.4	100	107	113	127 (118–134)
Sulfamethazine	Antibiotic	<0.12 (bdl)	<0.244 (bdl)	<0.165 (bdl)	<0.123 (bdl)	0.11 (0.07–0.17)
Erythromycin-H2O	Antibiotic	0.23	0.225	0.224	0.495	0.47 (0.23–0.66)
Flumequine	Antibiotic	<0.3 (bdl)	1.1	0.146	0.344	0.57 (0.16–1.37)
Diphenhydramine	Anticholinergic	<0.12 (bdl)	0.23	0.213	0.062	0.15 (0.06–0.25)
Sertraline	Antidepressant	<0.08 (bdl)	0.105	0.039	0.041	0.13 (0.04–0.24)
Venlafaxine	Antidepressant	<0.08 (bdl)	0.165	0.039	0.041	0.11 (0.04–0.26)
ΣAmitriptyline	Antidepressant	<0.06 (bdl)	0.06	0.07	0.05	0.07 (0.04–0.15)
Citalopram	Antidepressant	<0.08 (bdl)	0.1455	0.1625	0.041	0.10 (0.04–0.13)
ΣDiazepam	Antidepressant	0.91	0.81	0.39	0.41	0.89 (0.42–1.34)
ΣFluoxetine	Antidepressant	<0.3 (bdl)	1.18	0.73	0.32	0.92 (0.45–1.50)
Iopamidol	Contrast agent	<16 (bdl)	20.9	17.6	8.20	8.12 (7.85–8.35)
Gemfibrozil	Lipid regulator	<0.3 (bdl)	0.147	0.146	0.154	0.46 (0.15–0.86)
ΣCaffeine	Stimulant	<3.0 (bdl)	3.38	1.46	1.54	1.52 (1.47–1.57)
N,N-Diethyl-m-toluamide (DEET)	Repellent	0.235	0.416	0.41	0.314	0.46 (0.23–0.58)

The acronym “bdl” is short for below detection limit. ΣAmitriptyline = sum of amitriptyline and 10-hydroxy-amitriptyline; ΣCaffeine = sum of caffeine + 1,7-dimethylxanthine; ΣDiazepam = sum of diazepam and oxazepam; ΣFluoxetine = sum of fluoxetine and norfluoxetine. *likely present as a natural hormone (cortisol).

The bioaccumulation of the aforementioned drugs in fish indicates that, compared to the other drugs present in the waters of CPM, these drugs are either more bioavailable or not metabolized as quickly; or, are both more bioavailable and slowly metabolized. Being nearly 100% bioavailable in humans, gemfibrozil is an established cytochrome P450 (CYP450) inhibitor, and this inhibition is thought to reduce the metabolism of other drugs^22^. In the yellow European eel (*Anguilla anguilla*), CYP1A activity was inhibited 96 hr after injection with gemfibrozil^23^. Erythromycin and sulphonamides (such as sulfamethazine) are also known to inhibit CYP450 activity^24^. In zebrafish liver microsomes, a mixture of gemfibrozil, erythromycin, ciprofloxacin and fluoxetine inhibited CYP450-mediated reactions^25^. Gemfibrozil, fluoxetine, sulfamethazine, and erythromycin were detected in the plasma of our goldfish. Thus, we suspect the bioaccumulation of the PPCPS in fish from the present study was influenced by reduced metabolism, which might have been exasperated by inhibition of CYP450 phase-I metabolism by other drugs present in the mixture.

Reduced drug metabolism can cause drug-induced liver injury (DILI) in humans^24,26,27^. DILI accounts for over 50% of acute liver failure cases in the USA^24^. Anti-infectious agents, psychotropic drugs, and NSAIDs were among the most common culprits causing DILI at rates of 25%, 22.5%, and 10%, respectively, among all reported DILI cases over a 3-yr period in France^27^. The mechanisms of DILI generally involve mitochondrial dysfunction or induction/inhibition of cytochrome P450 isoenzymes^26^. In both cases, metabolic cholestasis can occur, resulting in an increase of cellular reactive oxygen species (ROS) and the loss of cellular glutathione (GSH) to reduced glutathione (GSSH) which can interfere with drug metabolism and clearance^26,28^. Although we did not directly measure ROS, GSH, or GSSH in this present study, the IPA software predicted that expression of glutathione would be activated, based upon the pattern of expression of 7 molecules that were measured in plasma of wild male goldfish from CPM (Table 1). Assuming the IPA prediction was correct, the wild male goldfish from CPM had increased levels of GSH in the liver, in a likely compensatory mechanism to help reduce oxidative stress caused by the impaired metabolism of drugs. Such compensatory mechanisms might also explain the ability of the goldfish caged closest to the Dundas WWTP effluent outfall (CPM1) to survive, despite the presence of expressed proteins and metabolites that are implicated in liver cell necrosis.

Gemfibrozil is designed to decrease accumulation of lipids by activating the peroxisome proliferator-activated receptor-alpha (PPARα), which increases the production of lipid metabolizing enzymes. It is possible that gemfibrozil exposure could have led to altered lipid levels in goldfish from CPM. Based upon expression levels of 13 proteins and metabolites, the IPA analysis predicted inhibition of lipid synthesis and accumulation in wild male goldfish (Table 1), which was supported by the observation of reduced plasma fatty acids, bile acids, and phosphatidylcholines compared to caged goldfish at the reference site (Fig. 5). Inhibited lipid synthesis was further supported by reduced bile acids and fatty acids in caged fish plasma. While concentrations of most fatty acids increased in goldfish caged closest to the WWTP outfall at CPM1, plasma fatty acids then decreased in fish caged further downstream at CPM2 and CPM3 when compared with goldfish caged at the reference site.

The reported effects of gemfibrozil on lipid metabolism in other teleost fish have been variable, but generally support our observations. For example, Skolness *et al*.^29^ observed increased triglycerides in female fathead minnow (*Pimephales promelas*) after short-term exposure (2d), reduced lipoprotein lipase (*lpl)* mRNA expression after an intermediate length of time (8d), and increased apolipoprotein A1 (*apoa1)* mRNA expression after longer term waterborne exposure (21d) at 600 mg gemfibrozil/L^29^. Whereas in male fathead minnow, *apoa1* mRNA expression was reduced (8d) and *lpl* mRNA expression was increased (2d)^29^. Prindiville *et al*.^30^ observed decreased plasma lipid levels and increased hepatic *lpl* mRNA expression in juvenile female rainbow trout (*Oncorhynchus mykiss*) following i.p. injection of 100 mg/kg of gemfibrozil every third day for 15 days^30^. In our study, expression of Apoa1 was increased and plasma fatty acids were decreased in wild goldfish (Figs 2 and 5) and fatty acids were also reduced in fish caged at CPM2 and CPM3 (Figs 1 and 4) at gemfibrozil plasma concentrations of 0.15 ng/g and water concentrations ranging from 4.75–41.3 ng/L. Thus, the changes in expression of lipid carrier proteins and lipid molecules that we observed in the goldfish from CPM seem to be typical for fish exposed to gemfibrozil in the laboratory albeit at much higher exposure concentrations (10^6^ times higher than the environmental bioaccumulation in the present study). It could be that goldfish are more sensitive to gemfibrozil than other species, or perhaps we observed a seasonal affect due to consumption of a different diet and nutritional status which may occur during summer months. However, the impact of environmental exposures to gemfibrozil on the long-term health and survival of fish in the wild remains unclear.

## Conclusions

The PPCPs that were detected in the plasma of caged and wild fish from CPM appear to have had subtle effects, occurring mostly at the molecular level. However, those molecular effects appear to have resulted in altered behaviour, which is discussed in detail in our companion manuscript^19^. The plasma metabolome and proteome responses in caged goldfish near the WWTP outfall at CPM2 and CPM1 most closely predicted the responses in wild goldfish. We observed changes in protein expression and metabolite concentrations that were suggestive of liver necrosis and altered lipid metabolism. These effects could have been caused by exposure to PPCPs present in WWTP effluents, but also could have been influenced by a broader set of pollutants which could also be present in CPM. Despite these apparently adverse indicators, survival was high in caged fish housed for three weeks along the wastewater effluent plume in Cootes Paradise. The expression of plasma metabolites and proteins in caged goldfish agreed well with those in the wild goldfish, suggesting that the combined use of ‘omic approaches and caged surrogates is a useful way to predict the molecular effects of contaminants in wild fish. Goldfish are known to be a highly resilient species, and as such, have proven highly successful as invaders of Great Lakes ecosystems^31^. Ultimately, the molecular responses we observed in these robust fish are likely conservative predictors of the potential effects of PPCPs and wastewater effluents on other wild fish species. Our findings suggest that future studies that focus on the mechanisms underlying metabolic disruption in fish exposed to wastewater effluents in the wild are warranted.

## Methods

All animal experiments were in accordance with CCAC guidance and approved by the GLLFAS-WSTD Animal Care Committee (Government of Canada).

### Wild goldfish collection

Wild goldfish were collected from CPM in May 2012, by electrofishing by Royal Botanical Gardens staff. Though multiple efforts were made to catch goldfish at the reference site and at other nearby locations where they were previously abundant, no wild goldfish were captured at JH in 2012. A map of the caging sites and the wild fish collection areas is shown in Fig. 1.

### Goldfish Caging

We purchased male goldfish in 2014 from AQUAlity Tropical Fish Wholesale, Inc. (Mississauga, ON) and housed the fish in 1500 L tanks with flow set for 1 L/g of fish/day in the Aquatic Life Research Facility (ALRF) (Environment Canada, Burlington, ON) for 2 weeks before deployment in the cages. Fish were formalin treated and fed with Northfin Goldfish Formula, Canadian Aquatic Feeds Ltd, Toronto at 2% of estimated bodyweight per day.

We constructed cages from Rubbermaid Hinged Top Totes (114 L, Polypropylene, Dimensions: 81 × 51.4 × 44.5 cm) with drilled holes that were 1.5875 cm in diameter. We modified each with stainless steel hardware to allow for suspension 30.5 cm above the sediment. Each cage housed 13 fish. We visited the cages weekly to feed the fish – 20 g of food per cage. The caged goldfish were deployed for 21 days from June25/26 – July 16/17, 2014. There were five cages at each of four sites, which are described in detail in our companion manuscript^19^. Briefly, three sites were located along the plume of the Dundas WWTP outfall in CPM: CPM1 (nearest to outfall), CPM2 (downstream from CPM1 and upstream of CPM3), and CPM3 (furthest from outfall), and the fourth site, JH, served as a reference site that was located outside of the CPM watershed but further south on Lake Ontario. JH was selected because as the control because we had previously collected water samples from there, and thus we knew the PPCP concentrations from that location (available in Muir *et al*.^20^). Additionally, because we could not capture any wild goldfish from JH in 2012, we elected to use fish caged at JH as a reference to assess the wild fish from CPM in 2012. We considered including a laboratory control, but there is evidence that variation in ‘Omics responses are much greater for field than laboratory exposures^21^, and thus laboratory held fish control might not provide a realistic negative control. Finally, we were confident that we could compare the wild goldfish captured in CPM in 2012 to goldfish caged at CPM in 2014, despite the temporal difference, because the accumulated levels of plasma PPCPs were remarkably similar (close to a factor of 1 and well within a factor of 2 in most cases) (see Table 2).

### Fish samples

We transported fish from the deployment sites back to the laboratory in bags of aerated, site-specific water. For both wild-captured and field caged fish, they were first anesthetized in an aerated solution of tricaine methanesulfonate (TMS; 50-60 mg/L) that was buffered with NaHCO_3_ (100-120 mg/L) (Animal Care Protocol AU1122) in a bath of water taken from each site. We collected blood from the caudal vein, and then separated plasma from blood using a refrigerated centrifuge into cryogenic vials as previously described^32^. Afterward, we immediately froze the plasma vials in liquid nitrogen, and we then stored the plasma at −80°C for future analyses. We then recorded mass and fork length of each fish, euthanized the fish by caudal vein severance and then excised and weighed the gonads from each fish.

### Vitellogenin

We measured plasma Vtg for 15 caged goldfish at CPM1 and for 15 caged goldfish at JH using an ELISA kit for carp Vtg (Biosense, Cedarlane Labs, Burlington, ON). Plasma was diluted 20x. The ELISA was calibrated against a Carp Vtg standard 62.5–0.06 ng/ml in 12 serial 1:1 dilutions.

### Proteomics

We thawed plasma samples from individual fish on ice and then transferred 15 μL of the plasma into a low-retention micro-centrifuge tube. We digested plasma proteins by formic acid digestion as previously described^32^. Next, we dried the digests to near dryness in a centrifugal evaporator, and then re-constituted the peptides in 20 μL of 95:5 Water:Acetonitrile with 0.1% formic acid. We injected 2 μL of the peptide solution and then performed a separation by reverse phase liquid chromatography on a Zorbax, 300SB-C18, 1.0 × 50 mm 3.5 μm column (Agilent Technologies Canada Inc., Mississauga, ON) using an Agilent 1260 Infinity Binary LC^32^. The Agilent 6520 Accurate-Mass Quadrupole Time of- Flight (Q-TOF) was used as the detector in tandem to the Agilent 1260 system^33^. Each analytical run included a solvent blank, peptide standard (H2016, Sigma-Aldrich, Oakville, ON), and a BSA digest standard (Agilent Technologies Canada Inc, Mississauga, ON) injection every 10 samples in order to monitor baseline, carry-over, drift, and sensitivity during the runtime. We injected once per individual sample.

We identified proteins by search against the National Center for Biotechnology Information (NCBI) Teleostei (teleost fishes) protein database (downloaded March 4, 2015) as previously described^10^. Spectral files for each fish (n = 25) were pooled into groups by location. Each group was analyzed separately using Spectrum Mill Software (Version B.04.01.141). We manually validated and accepted a protein when at least one peptide had a peptide score (quality of the raw match between the observed spectrum and the theoretical spectrum) greater than 5 and a %SPI (percent of the spectral intensity that are accounted for by theoretical fragments) of greater than 60% (these setting are recommended by the manufacturer for validating results obtained with an Agilent Q-TOF mass spectrometer).

### Metabolomics

We analyzed the plasma metabolome from individual gold fish (2012 field-collected and 2014 caged). AXYS Analytical Services, Ltd (Sidney, BC, CA) carried out the analysis using a targeted metabolomics platform^34^ with modifications. The platform contained a total of 217 metabolites including 21 amino acids (AA), 23 biogenic amines (BAs), 13 bile acids, ∑hexose, 15 fatty acids (FAs), 40 acylcarnitines (ACs), 90 phosphatidylcholines (PCs), and 15 sphingomyelines (SMs) were measured. We provide a full list of analytes, internal standards and abbreviations in Supplemental Materials Table S4.

We added each sample (10 µl of goldfish plasma for AA and BA or 50 µl for all other metabolites) to a 96-well filter plate (Pall Corporation, Port Washington, NY, USA) that was fortified with an internal standard mix (Table S5). We then dried the plates were under liquid nitrogen, and we derivatized the AAs and BAs using Edman’s Reagent^35^. After drying, we added 250 μL of 5 mM ammonium acetate in methanol to each well, and the plate was shaken for 30 min. We eluted the samples into a Nunc 96-deep well plate (Thermo Scientific, Waltham, MA, USA) by centrifugation (100 g for 2 min at ambient temperature) and diluted with an equivalent volume of water (methanol for ACs, PCs, and SM) prior to analysis.

We measured the concentrations of metabolites using an Agilent 1100 high performance liquid chromatography (HPLC) system (Agilent, Palo Alto, California, USA) coupled to an API4000 triple quadrupole mass spectrometer (Applied Biosystems/Sciex, Concord, ON, Canada). AAs and BAs were analyzed as phenylthiocarbamyl derivatives. ΣHexose, FAs and bile acids were analyzed separately by HPLC−MS/MS. All these analytes were quantified by isotope dilution/surrogate quantification using a 5–7 calibration curve generated from authentic native standards. ACs, SMs and PCs were measured using flow-injection MS/MS (FI-MS/MS). After deconvolution of overlapping isotopic peaks^36^, we quantified the lipid analytes relative to an internal standard. Mean method detection limits for each target metabolite are available in Table S6.

We processed and analyzed three blanks and three internal reference human serum samples (MP Biomedicals, Santa Ana, California, USA) with each batch of samples. We used the blanks to estimate background concentrations of metabolites during sample workup, and the reference samples to estimate analytical precision through sample workup. In addition, we ran a calibration sample every 20 samples to assess instrument stability, and we ran instrument methanol blank samples after high concentration calibration samples to assess sample carryover on the instrument. We previously validated the method at two different spiking levels (n = 5) in human plasma and then verified the method for goldfish plasma by analysing different sample amounts to assess appropriate sample size and to assess potential for interferences specific to goldfish plasma.

### Statistical and Bioinformatics Analyses

For fish biometrics (Liver somatic index, LSI; Gonadal somatic index, GSI; and Condition Factor, K), and Vtg, we visually examined the data using box-whisker plots, and then tested to see if they met the assumptions of normality using Statistix 10. We verified agreement with the assumptions of normality and homogeneity of variance using the Shapiro-Wilk statistic, Levene test, O’Briens’s test, and Brown and Forsythe test (one-way analysis of variance). When data did not conform to these assumptions of ANOVA, we adjusted the data using logarithmic transformations and when transformed data still did not meet those assumptions, we tested for significant difference using the non-parametric Kruskal-Wallis test.

We used the NCBI non-redundant database to match valid protein IDs to the closest human protein ortholog using the protein BLAST tool so that we could use the corresponding human gene symbol for functional analysis. In cases where there was more than one peptide or set of peptides matched to the same protein (this can happen when different peptides are matched to the same protein for different species in the database), we consolidated the data manually using Excel to calculate new mean intensities, number of peptides, and percent protein coverage; we selected the lowest FDR and highest SPI values to represent the quality of these consolidated protein IDs. We included single peptide IDs if their FDR was <1%.

We manually searched for and then matched metabolites to Human Metabolome Database (HMDB) numbers for functional analysis. We used Metaboanalyst 3.0 to calculate all fold change values and to perform Analysis of Variance (ANOVAs) with Fisher’s LSD post-hoc test to determine differences in protein and metabolite data between sites. We retained the Metaboanalyst default settings for metabolite concentration data, while median normalization with pareto-scaling was selected for protein peak intensity data. We used the metabolite and protein data from male goldfish caged at the reference site (JH) as the reference for fold change calculations on the wild male goldfish data.

We used QIAGEN’s Ingenuity® Pathway Analysis (IPA) software (QIAGEN Redwood City, www.qiagen.com/ingenuity) to determine the biological functions for both metabolite and protein IDs together (core analysis). We uploaded pooled data for each location into the application, with corresponding human gene symbol or HMDB identifiers and fold change values based upon comparison to the reference site (JH). IPA mapped each identifier to its corresponding object in Ingenuity’s Knowledge Base. IPA overlaid these molecules, called network eligible molecules, onto a global molecular network developed from information contained in Ingenuity’s Knowledge Base, and then algorithmically generated functional networks based on their connectivity. Core analysis identified the biological functions and/or diseases that were most significant to the data set. IPA used the right-tailed Fisher’s Exact Test to calculate a *p*-value determining the probability that each biological function assigned to that data set is due to chance alone. IPA also calculated the overlap p-value using the one-sided Fisher’s Exact Test as a measure of the enrichment of the dataset (i.e. how much of the dataset overlaps with the known regulators in Ingenuity’s Knowledge Base). Finally, the IPA software calculated an activation z-score for each biological function which takes into account the predicted direction of expression (based upon the Ingenuity Knowledge Base) versus the observed direction of expression within the dataset to infer whether the function is activated (z-score >+2) or inhibited (z-score <−2).

### Data availability

All data generated or analysed during this study are included in this published article and in Supplementary Information

## Electronic supplementary material

Supplementary Information
