# Supplementary material for: Altered expression of metabolites and proteins in wild and caged fish exposed to wastewater effluents in situ
Source: Sci Rep. 2017 Dec 5;7:17000. doi: 10.1038/s41598-017-12473-6 (PMC5717254; doi:10.1038/s41598-017-12473-6)
Supplement: Supplementary file 1 — Supplementary Information [file 41598_2017_12473_MOESM1_ESM.pdf]

Supplementary Information

Altered expression of metabolites and proteins in wild and caged fish exposed to wastewater effluents in situ.

Simmons D1, Miller J[1], Clarence S[1], McCallum E[2], Balshine S[2], Chandramouli B[3], Cosgrove J[3], Sherry J[1*].

[1] Aquatic Contaminants Research Division, Water Science and Technology Directorate, Environment and Climate Change Canada, Burlington, ON

[2] Department of Psychology, Neuroscience & Behaviour, McMaster University, Hamilton, ON

[3] Metabolomics Services, SGS AXYS, Sidney, BC

[*]corresponding author

Supplementary Table 1: Morphometric Data

	Wild Goldfish (2012)	Caged Goldfish (2014)			
**Location**	**CPM**	**CPM1**	**CPM2**	**CPM3**	**JH**
**N**	**20**	**25**	**25**	**25**	**25**
Mean LSI (Standard Error)	1.808 (0.0757)	not recorded			
Mean GSI (Standard Error)	3.628 (0.1876)	2.789 (0.2186)	2.786 (0.202)	2.579 (0.1873)	3.08 (0.2811)
Mean K (Standard Error)	1.906 (0.04027)	1.735 (0.04768)	1.815 (0.02868)	1.687 (0.03574)	1.617 (0.04027)

Supplementary Table 2. Detailed protein search results

Caged Goldfish														
			CPM1			CPM2			CPM3			JH		
symbol	Accession Number	Database	# peptides	Search Score	% protein coverage	# peptides	Search Score	% protein coverage	# peptides	Search Score	% protein coverage	# peptides	Search Score	% protein coverage
Aftph	CDQ90298.1	NCBI	1	9.84	0.7	1	10.36	0.7	1	10.38	0.7			
Ankrd12	XP_005751611	NCBI	1	5.21	1.7							1	6.34	1.7
Ash1l	XP_003966329	NCBI				1	6.41	2.4	2	15.02	2.5	3	21.02	2.7
Atad2b	AAW82445.1	NCBI	19	208.12	50.3	21	218.73	53.9	23	231.31	58.1	24	243.63	53.9
Brd1	XP_698063.5	NCBI	5	38.92	5.6									
Btd	XP_004073869	NCBI				1	6.32	1.4	1	5.75	1.4	1	6.82	1.4
C3	BAA36618.1	NCBI	15	108.76	10.2	4	33.6	2	11	75.29	7.1	11	72.54	8.5
Chmp6	ACO09124.1	NCBI	1	7.35	6.2	1	6.43	6.2	1	5.47	6.2			
Cracr2a	XP_005805973	NCBI	1	7.13	4	1	8.39	4	1	7.42	4	1	9.3	4
Cyp51a1	CDQ67700.1	NCBI				1	6.04	2.1				1	6.49	2.1
Daam1	XP_004541887	NCBI	1	6.81	0.8	1	7.13	0.8	1	6.23	0.8	1	8.29	0.8
Dab2ip	XP_003976523	NCBI							1	5.27	1.9	1	6.77	2
Epdr1	AAB40068.1	NCBI	1	6.05	17.1	1	8.88	17.1	1	9.1	17.1	2	13.8	23.2
Epm2a	CDQ80564.1	NCBI	1	5.73	10.4	1	6.4	10.4	1	5.06	10.4			
Fetub	ABA33614.1	NCBI	2	19.61	4.8	1	14.4	1.4	2	18.03	9.6	1	13.54	1.4
Fgg	ABD83891.1	NCBI	11	84.26	29.5	6	53.32	16.7	10	84.85	54.1	7	59.12	15.6
Fn1	AAU14809.1	NCBI	9	60.2	5.1	1	8.2	0.8				1	7.41	0.8
Hbb	P02140.1	NCBI	12	133.67	53.7	8	82.89	33.3	9	99.45	47.6			
Hbe1	0606173B	NCBI										10	102.52	47.6
Hpx	BAD98538.1	NCBI	18	190.26	31	22	231.17	36.7	26	267.98	45.2	13	158.07	25.3
Ifi44	AAP20189.1	NCBI	1	5.91	11.8	1	5.59	11.8	1	5.27	11.8			
Iglc6	BAB90987.1	NCBI	5	72.81	48.3	4	54.33	37.5	4	73.94	37.5	4	72.99	37.5
Il10rb	ABJ97307.1	NCBI	1	5.63	4.9	1	9.27	4.9						
Myo5c	CAG05565.1	NCBI	1	9.66	1.7	1	7.69	1.7				1	7.87	1.7
Nphs1	CAG12048.1	NCBI	1	8.43	0.7	1	5.97	0.7	1	7.14	0.7	1	6.7	0.7
Or52k1	CAG09001.1	NCBI	1	8.31	1.9	1	9.09	1.9	1	8.33	1.9	1	8.23	1.9
Psme4	XP_004077490	NCBI	1	8.12	0.3	1	7.85	0.3	1	8.95	0.3	1	7.64	0.3
Serpina1	AAA73954.1	NCBI	6	45.42	14.7	4	34.06	9.9	6	53.83	16.5	12	105.23	21.9
Serpina5	AGO58874.1	NCBI	4	33.53	15	8	67.08	36.6	3	29.98	15	3	30.41	15
Smyd2	DAA01312.1	NCBI	1	11.03	3	1	7.09	3	1	6.38	3			
Snrnp25	XP_003442116	NCBI	1	6.5	5.8	1	6.04	5.8	1	8.69	5.8	1	8.92	5.8
Sptbn1	CAG13137.1	NCBI	1	5.94	0.4	1	6	0.4						
Taf2	CAF95588.1	NCBI	1	5.82	1.7							1	6.24	1.7
Tf	P80426.1	NCBI	27	238.83	30.7	25	206.81	29.1	23	198.25	26.8	11	101.04	17.2
Usp39	XP_003975010	NCBI	1	6.65	1.8	1	6.39	1.8	1	6.23	1.8	1	5.24	1.8
Znf500	CAG00059.1	NCBI	1	5.33	0.4	1	7.72	0.4				1	6.81	0.4

Supplementary Table 3. Single Peptide ID search details

CPM1

accession number	file number	parent charge	score	Local FDR (%)	Global FDR (%)	n-terminus	previous aa	sequence	next aa	c-terminus	modifications	Retention Time (Min)	Chromatographic Peak Width (Sec)	Parent m/z	Matched Parent Mass
998316	1427	4	6.05	0.58	0.28	Hydrogen	(D)	DAIAWGEFKYDSSQKHLRFVEDTGKSNKTSYLD	(V)	Free Acid	0	18.78	16.31	959.7313	3835.857
32140151	480	3	11.03	0.08	0.02	Hydrogen	(D)	EELSHLGSAIFPD	(V)	Free Acid	0	12.3	15.24	472.2316	1414.685
37779058	1492	3	5.91	0.58	0.3	Hydrogen	(D)	LGIPQMAIITNID	(G)	Free Acid	0	13.45	16.9	466.9268	1398.767
47220852	1735	2	5.33	0.64	0.38	Hydrogen	(D)	VATRYDGLD	(H)	Free Acid	0	1.9	9.43	505.2484	1009.495
47225565	845	2	8.43	0.24	0.09	Hydrogen	(D)	FTHPPVD	(S)	Free Acid	0	4.48	26.11	406.7012	812.394
47227838	856	2	8.31	0.24	0.1	Hydrogen	(D)	IKQTLI	(-)	Free Acid	0	10.8	8.89	358.2371	715.472
51949771	1519	5	5.86	0.34	0.07	Hydrogen	(D)	ITGYRVTGTPINGQRGVSLEESVRGDETSCILE	(N)	Free Acid	0	12.72	3.37	708.1539	3536.766
75991522	187	2	14.29	0.01	0.01	Hydrogen	(D)	PLFKPTV	(-)	Free Acid	0	11.9	15.04	401.2499	801.487
225706556	1047	3	7.35	0.3	0.14	Hydrogen	(D)	AVLAELELITQGD	(V)	Free Acid	0	14.23	21.93	457.9254	1371.737
348509153	1275	2	6.5	0.5	0.21	Hydrogen	(D)	EDALPHSEILD	(I)	Free Acid	0	3.18	24.68	619.798	1238.591
548524821	1827	3	5.21	0.64	0.4	Hydrogen	(D)	VPLFTNGFKYQD	(V)	Free Acid	0	2.65	28.9	476.9149	1428.716
551508890	1097	5	7.13	0.31	0.16	Hydrogen	(D)	VFCAIPRPSLRKKQMEGLADLFDDESQPAKRD	(P)	Free Acid	0	14	3.06	732.9718	3660.863
642093759	1572	6	5.73	0.58	0.32	Hydrogen	(D)	VHLAEPCQESLWLKFIKRVGGNFIWEGNGSHHD	(R)	Free Acid	0	14	9.89	634.8213	3803.887

CPM2

accession number	file number	parent charge	score	Local FDR (%)	Global FDR (%)	n-terminus	previous aa	sequence	next aa	c-terminus	modifications	Retention Time (Min)	Chromatographic Peak Width (Sec)	Parent m/z	Matched Parent Mass
998316	726	4	8.88	0.2	0.05	Hydrogen	(D)	DAIAWGEFKYDSSQKHLRFVEDTGKSNKTSYLD	(V)	Free Acid	0	18.82	7.83	959.7353	3835.857
32140151	1096	3	7.09	0.34	0.14	Hydrogen	(D)	EELSHLGSAIFPD	(V)	Free Acid	0	12.42	14.49	472.2322	1414.685
37779058	1647	3	5.59	0.53	0.31	Hydrogen	(D)	LGIPQMAIITNID	(G)	Free Acid	0	13.42	18.87	466.9297	1398.767
47220852	953	2	7.72	0.27	0.1	Hydrogen	(D)	VATRYDGLD	(H)	Free Acid	0	1.95	4.94	505.253	1009.495
47225565	1477	2	5.97	0.51	0.26	Hydrogen	(D)	FTHPPVD	(S)	Free Acid	0	4.48	29.63	406.6978	812.394
47227838	683	2	9.09	0.16	0.05	Hydrogen	(D)	IKQTLI	(-)	Free Acid	0	10.83	14.29	358.237	715.472
75991522	170	2	14.4	0.01	0.01	Hydrogen	(D)	PLFKPTV	(-)	Free Acid	0	11.97	14.99	401.2454	801.487
225706556	1266	3	6.43	0.44	0.2	Hydrogen	(D)	AVLAELELITQGD	(V)	Free Acid	0	14.22	17.95	457.926	1371.737
348509153	1426	2	6.04	0.51	0.26	Hydrogen	(D)	EDALPHSEILD	(I)	Free Acid	0	3.2	17.52	619.8018	1238.591
410905699	1274	5	6.41	0.44	0.2	Hydrogen	(D)	DEAPSQPVAFQREEKLDGPPRKKYLVAGLYSD	(D)	Free Acid	0	18.22	3.18	721.5648	3603.845
432881621	1309	2	6.32	0.5	0.21	Hydrogen	(D)	FVLEGRFD	(T)	Free Acid	0	4.62	23.63	491.7447	982.5
551508890	812	5	8.39	0.2	0.08	Hydrogen	(D)	VFCAIPRPSLRKKQMEGLADLFDDESQPAKRD	(P)	Free Acid	0	13.93	2.33	732.9755	3660.863
642093759	1281	5	6.4	0.44	0.21	Hydrogen	(D)	VHLAEPCQESLWLKFIKRVGGNFIWEGNGSHHD	(R)	Free Acid	0	14.38	8.54	761.5825	3803.887

CPM3

accession number	file number	parent charge	score	Local FDR (%)	Global FDR (%)	n-terminus	previous aa	sequence	next aa	c-terminus	modifications	Retention Time (Min)	Chromatographic Peak Width (Sec)	Parent m/z	Matched Parent Mass
998316	831	4	9.1	0.16	0.05	Hydrogen	(D)	DAIAWGEFKYDSSQKHLRFVEDTGKSNKTSYLD	(V)	Free Acid	0	18.8	12.33	959.7208	3835.857
32140151	1402	2	6.38	0.46	0.16	Hydrogen	(D)	EELSHLGSAIFPD	(V)	Free Acid	0	12.43	27.77	707.8482	1414.685
37779058	1871	3	5.27	0.62	0.32	Hydrogen	(D)	LGIPQMAIITNID	(G)	Free Acid	0	13.4	16.24	466.9307	1398.767
47225565	1194	2	7.14	0.24	0.11	Hydrogen	(D)	FTHPPVD	(S)	Free Acid	0	4.38	27.72	406.6987	812.394
47227838	970	2	8.33	0.16	0.07	Hydrogen	(D)	IKQTLI	(-)	Free Acid	0	10.8	14.69	358.2376	715.472
75991522	366	2	12.44	0.04	0.004	Hydrogen	(D)	PLFKPTV	(-)	Free Acid	0	11.98	15	401.2491	801.487
225706556	1781	3	5.47	0.62	0.28	Hydrogen	(D)	AVLAELELITQGD	(V)	Free Acid	0	14.22	35.43	457.9264	1371.737
348509153	892	2	8.69	0.16	0.06	Hydrogen	(D)	EDALPHSEILD	(I)	Free Acid	0	3.18	24.3	619.8096	1238.591
410905699	999	5	8.17	0.16	0.07	Hydrogen	(D)	DEAPSQPVAFQREEKLDGPPRKKYLVAGLYSDD	(Y)	Free Acid	0	17.78	8.88	744.5708	3718.872
432881621	1643	2	5.75	0.53	0.24	Hydrogen	(D)	FVLEGRFD	(T)	Free Acid	0	4.73	24.22	491.7442	982.5
551508890	1139	5	7.42	0.23	0.1	Hydrogen	(D)	VFCAIPRPSLRKKQMEGLADLFDDESQPAKRD	(P)	Free Acid	0	13.57	3.15	732.9758	3660.863
642093759	1980	6	5.06	0.62	0.35	Hydrogen	(D)	VHLAEPCQESLWLKFIKRVGGNFIWEGNGSHHD	(R)	Free Acid	0	14.47	3.34	634.8147	3803.887

Supplementary Table 3. Single Peptide ID search details cont.

JH

accession number	file number	parent charge	score	Local FDR (%)	Global FDR (%)	n-terminus	previous aa	sequence	next aa	c-terminus	modifications	Retention Time (Min)	Chromatographic Peak Width (Sec)	Parent m/z	Matched Parent Mass
998316	894	4	8.26	0.28	0.07	Hydrogen	(D)	DAIAWGEFKYDSSQKHLRFVEDTGKSNKTSYLD	(V)	Free Acid	0	18.85	9.94	959.7337	3835.857
47220852	1200	2	6.81	0.34	0.16	Hydrogen	(D)	VATRYDGLD	(H)	Free Acid	0	1.98	4.53	505.246	1009.495
47225565	1230	2	6.7	0.34	0.17	Hydrogen	(D)	FTHPPVD	(S)	Free Acid	0	4.42	21.95	406.7009	812.394
47227838	897	2	8.23	0.28	0.07	Hydrogen	(D)	IKQTLI	(-)	Free Acid	0	10.68	14.15	358.24	715.472
75991522	244	2	13.54	0.01	0.005	Hydrogen	(D)	PLFKPTV	(-)	Free Acid	0	11.98	16.13	401.2488	801.487
348509153	776	2	8.92	0.19	0.04	Hydrogen	(D)	EDALPHSEILD	(I)	Free Acid	0	3.48	17.76	619.806	1238.591
410905699	1015	5	7.6	0.31	0.1	Hydrogen	(D)	DEAPSQPVAFQREEKLDGPPRKKYLVAGLYSD	(D)	Free Acid	0	18.15	5.72	721.5652	3603.845
410926113	1209	3	6.77	0.34	0.17	Hydrogen	(D)	NVQRTENSLSLWVNEAKD	(L)	Free Acid	0	12.72	5.32	701.69	2103.047
432881621	1198	2	6.82	0.34	0.16	Hydrogen	(D)	FVLEGRFD	(T)	Free Acid	0	4.5	27.12	491.7447	982.5
548524821	1342	3	6.34	0.46	0.19	Hydrogen	(D)	VPLFTNGFKYQD	(V)	Free Acid	0	2.68	3.93	476.9149	1428.716
551508890	710	5	9.3	0.06	0.03	Hydrogen	(D)	VFCAIPRPSLRKKQMEGLADLFDDESQPAKRD	(P)	Free Acid	0	13.93	5.05	732.9914	3660.863

Wild Goldfish CPM

accession number	file number	parent charge	score	Local FDR (%)	Global FDR (%)	n-terminus	previous aa	sequence	next aa	c-terminus	modifications	Retention Time (Min)	Chromatographic Peak Width (Sec)	Parent m/z	Matched Parent Mass
M3ZEN5	749	3	6.22	0.34	0.12	Hydrogen	(D)	HPDLGPTEKITKYCD	(K)	Free Acid	0	13.45	4.32	572.9462	1716.827
XP_00407622[illegible]	805	3	6	0.20	0.14	Hydrogen	(D)	SRYSVKYSAPRSAATSYKEQNYI	(-)	Free Acid	0	17.28	9.67	890.4632	2669.332
A0A087XDY8	955	5	5.44	0.65	0.60	Hydrogen	(-)	MSFQVKKRPAGDCLLIKGGKVVNDD	(Q)	Free Acid	0	19.83	6.93	544.4928	2718.443
W5KRN7	811	3	5.97	0.65	0.55	Hydrogen	(D)	LAQRRGSVRD	(V)	Free Acid	0	16.4	20.38	386.5607	1157.65
XP_00397203[illegible]	352	3	9.75	0.01	0.01	Hydrogen	(D)	ILQASDHLSHAINSYEKIVD	(G)	Free Acid	0	14.08	10.85	751.7304	2253.151
M4AL52	1136	5	6.95	0.50	0.62	Hydrogen	(D)	KIVKRNNGEHVKCRKLEDAATFPSADD	(P)	Free Acid	0	14.08	29.13	609.1164	3041.559
H3CB65	734	3	6.27	0.65	0.54	Hydrogen	(D)	LAPRLPQLPD	(C)	Free Acid	0	11.47	14.61	373.8934	1119.653
I3KSB1	699	2	6.44	0.65	0.45	Hydrogen	(D)	ILQALVLTD	(R)	Free Acid	0	11.97	14.09	493.3029	985.593
H2LD28	591	5	7.12	0.59	0.33	Hydrogen	(D)	DKAQRLMKAKKLDSDEIDNKEEGEGSGKRKAED	(D)	Free Acid	0	18.38	35.96	744.5774	3718.867
AGO58874.1	661	3	6.7	0.27	0.08	Hydrogen	(D)	MMKRTGRYEIYQD	(P)	Free Acid	0	6.57	25.69	564.2641	1690.805
CAF99960.1	1554	3	5.75	0.83	0.51	Hydrogen	(D)	LGSDRHSCFKIKD	(Y)	Free Acid	0	6.55	31.27	502.5989	1505.754
ABD85567.1	535	4	7.54	0.21	0.04	Hydrogen	(D)	RRDDDSFVSAVAKSLFAD	(G)	Free Acid	0	9.98	31.79	500.4995	1998.988
G3NSD1	1261	4	5.55	0.70	0.63	Hydrogen	(D)	DSISKSTQFHDKID	(P)	Free Acid	0	5.18	29.19	405.9523	1620.787
XP_005744222	1404	6	5.11	0.68	0.44	Hydrogen	(D)	LNESREGGACRSQAQPISSQNHQNAGGSAPKPKQ	(E)	Free Acid	0	18.53	5.82	625.3174	3746.802
H2M560	695	2	6.47	0.65	0.42	Hydrogen	(D)	YIPLRD	(L)	Free Acid	0	6.32	32.88	388.7151	776.431
G3PDG5	917	3	5.56	0.65	0.63	Hydrogen	(D)	CHIALYEHMISKD	(T)	Free Acid	0	3.25	8.8	520.5825	1559.735
CDQ89122.1	807	4	5.98	0.52	0.14	Hydrogen	(D)	LALLRLPSPKD	(H)	Free Acid	0	10.3	19.71	306.441	1222.752
XP_00407749[illegible]	1058	2	5.19	0.60	0.27	Hydrogen	(D)	YIPLRD	(L)	Free Acid	0	6.07	35.26	388.7169	776.431
H2U1L5	691	5	6.49	0.65	0.44	Hydrogen	(D)	STKKHPYSVTVAMVFGRAVSTVFSTWLSEYPED	(F)	Free Acid	0	18.7	6.53	744.7742	3719.842
W5U6T2	582	4	7.16	0.59	0.35	Hydrogen	(D)	GPLSRHYRHYIGIMAAARHQCSYLVNLHVND	(F)	Free Acid	0	21.02	58.06	898.7141	3591.797
W5LDR9	681	5	6.57	0.59	0.44	Hydrogen	(D)	CNPFNDKQAEDRCHRMGQTRSVQVIKLVSKD	(T)	Free Acid	0	18.72	0	721.3654	3602.774
G3P736	472	2	8.21	0.3	0.33	Hydrogen	(D)	LGAIEEKYD	(V)	Free Acid	0	10.83	36.25	519.2653	1037.516
XP_004546934	951	3	5.45	0.60	0.22	Hydrogen	(D)	RETYPKYPEDPEDPKD	(P)	Free Acid	0	4.77	26.8	660.2988	1978.903
NP_001098244	1005	5	5.32	0.6	0.25	Hydrogen	(D)	FVFRCNIPPIKEKYSTDVGSKTD	(L)	Free Acid	0	15.9	3.1	529.6655	2644.345
F1RCR6.1	384	5	9.37	0.08	0.006	Hydrogen	(D)	DEANPLGRAGIWTKTHTVWYKPVREDEEGCKD	(A)	Free Acid	0	17.13	10.22	740.9526	3700.782
XP_002664661	1037	3	5.25	0.6	0.26	Hydrogen	(D)	FVLVPHLSGEQSYD	(L)	Free Acid	0	8.5	20.05	530.9368	1590.78
XP_004555233	561	2	7.29	0.21	0.05	Hydrogen	(D)	MKFPEIED	(A)	Free Acid	0	11.65	18.64	504.7433	1008.471
A0A087YFD3	606	4	7	0.59	0.42	Hydrogen	(D)	IASGDVSRKKYIFENQTSD	(I)	Free Acid	0	16.28	15.08	540.2835	2158.078

Supplementary Table 4. Panel of Metabolite Targets

List 1-Amino Acids and Biogenic amines

LC-MS/MS Analysis

#	Sub-Class	Name	Abbreviation	Surrogate
1	Amino Acid	Alanine	Ala	d4-Ala
2	Amino Acid	Arginine	Arg	15N2-Arg
3	Amino Acid	Asparagine	Asn	15N2-Asn
4	Amino Acid	Aspartate	Asp	d3-Asp
5	Amino Acid	Citrulline	Cit	13C-d4-Cit
6	Amino Acid	Glutamine	Gln	d5-Gln
7	Amino Acid	Glutamate	Glu	d3-Glu
8	Amino Acid	Glycine	Gly	13C2-15N-Gly
9	Amino Acid	Histidine	His	13C6-His
10	Amino Acid	Isoleucine	Ile	13C6-Ile
11	Amino Acid	Leucine	Leu	13C6-Ile
12	Amino Acid	Lysine	Lys	d6-Orn
13	Amino Acid	Methionine	Met	d3-Met
14	Amino Acid	Ornithine	Orn	d6-Orn
15	Amino Acid	Phenylalanine	Phe	d5-Phe
16	Amino Acid	Proline	Pro	d7-Pro
17	Amino Acid	Serine	Ser	d3-Ser
18	Amino Acid	Threonine	Thr	13C4-Thr
19	Amino Acid	Tryptophan	Trp	15N2-Trp
20	Amino Acid	Tyrosine	Tyr	d4-Tyr
21	Amino Acid	Valine	Val	d8-Val
22	Biogenic Amine	Acetylornithine	Ac-Orn	d6-Orn
23	Biogenic Amine	Asymmetric dimethylarginine	ADMA	d7-ADMA
24	Biogenic Amine	Symmetric dimethylarginine	SDMA	d7-ADMA
25	Biogenic Amine	Total dimethylarginine	total DMA	d7-ADMA
26	Biogenic Amine	alpha-Aminoadipic acid	alpha-AAA	d6-Orn
27	Biogenic Amine	Carnosine	Carnosine	13C6-His
28	Biogenic Amine	Creatinine	Creatinine	d3-Creatinine
29	Biogenic Amine	3-hydroxytyrosine	DOPA	d3-DOPA

30	Biogenic Amine	Dopamine	Dopamine	d4-Dopamine
31	Biogenic Amine	Histamine	Histamine	13C6-His
32	Biogenic Amine	Gamma-aminobutyric acid	GABA	d8-Val
33	Biogenic Amine	Kynurenine	Kynurenine	d4-Tyr
34	Biogenic Amine	Methioninesulfoxide	Met-SO	d3-Met
35	Biogenic Amine	Nitrotyrosine	Nitro-Tyr	d4-Tyr
36	Biogenic Amine	Hydroxyproline	OH-Pr	d7-Pro
37	Biogenic Amine	Phenylethylamine	PEA	d4-Serotonin
38	Biogenic Amine	Putrescine	Putrescine	d4-Putrescine
39	Biogenic Amine	Sarcosine	Sarcosine	d3-Sarcosine
40	Biogenic Amine	Serotonin	Serotonin	d4-Serotonin
41	Biogenic Amine	Spermidine	Spermidine	d4-Putrescine
42	Biogenic Amine	Spermine	Spermine	d4-Putrescine
43	Biogenic Amine	Taurine	Taurine	13C2-Taurine

List 2-Sugars
(LC-MS/MS-Analysis)

#	Sub-Class	Name	Abbreviation	Surrogate
44	Sugar	Hexose (sum isomers)	Hex	13C6 Glucose

List 3-Acylcarnitines, Glycerophospholipids, Sphingolipids
(FI-MS/MS Analysis)

#	Sub-Class	Name	Abbreviation	Surrogate
45	Acylcarnitine	Carnitine	AC C0	d9-AC C0
46	Acylcarnitine	Acetylcarnitine	AC C2	d3-AC C2
47	Acylcarnitine	Propionylcarnitine	AC C3	d3-AC C3
48	Acylcarnitine	Propenoylcarnitine	AC C3:1	d3-AC C3
49	Acylcarnitine	Hydroxypropionylcarnitine	AC C3-OH	d3-AC C3
50	Acylcarnitine	Butyrylcarnitine	AC C4	d3-AC C4
51	Acylcarnitine	Butenylcarnitine	AC C4:1	d3-AC C4
52	Acylcarnitine	Hydroxylbutyrylcarnitine	AC C4-OH (C3-DC)	d3-AC C4
53	Acylcarnitine	Valerylcarnitine	AC C5	d9-AC C5
54	Acylcarnitine	Tiglylcarnitine	AC C5:1	d9-AC C5
55	Acylcarnitine	Glutaconylcarnitine	AC C5:1-DC	d9-AC C5
56	Acylcarnitine	Glutarylcarnitine (Hydroxyhexanoylcarnitine)	AC C5-DC (C6-OH)	d9-AC C5
57	Acylcarnitine	Methylglutarylcarnitine	AC C5-M-DC	d9-AC C5

58	Acylcarnitine	Hydroxyvalerylcarnitine (Methylmalonylcarnitine)	AC C5-OH (C3-DC-M)	d9-AC C5
59	Acylcarnitine	Hexanoylcarnitine (Fumarylcarnitine)	AC C6 (C4:1-DC)	d9-AC C5
60	Acylcarnitine	Hexanoylcarnitine	AC C6:1	d9-AC C5
61	Acylcarnitine	Pimelylcarnitine	AC C7-DC	d3-AC C8
62	Acylcarnitine	Octanoylcarnitine	AC C8	d3-AC C8
63	Acylcarnitine	Nonaylcarnitine	AC C9	d3-AC C8
64	Acylcarnitine	Decanoylcarnitine	AC C10	d3-AC C8
65	Acylcarnitine	Decenoylcarnitine	AC C10:1	d3-AC C8
66	Acylcarnitine	Decadienylcarnitine	AC C10:2	d3-AC C8
67	Acylcarnitine	Dodecanoylcarnitine	AC C12	d9-AC C14
68	Acylcarnitine	Dodecenoylcarnitine	AC C12:1	d9-AC C14
69	Acylcarnitine	Dodecanedioylcarnitine	AC C12-DC	d9-AC C14
70	Acylcarnitine	Tetradecanoylcarnitine	AC C14	d9-AC C14
71	Acylcarnitine	Tetradecenoylcarnitine	AC C14:1	d9-AC C14
72	Acylcarnitine	Hydroxytetradecenoylcarnitine	AC C14:1-OH	d9-AC C14
73	Acylcarnitine	Tetradecadienylcarnitine	AC C14:2	d9-AC C14
74	Acylcarnitine	Hydroxytetradecadienylcarnitine	AC C14:2-OH	d9-AC C14
75	Acylcarnitine	Hexadecanoylcarnitine	AC C16	d3-AC C16
76	Acylcarnitine	Hexadecenoylcarnitine	AC C16:1	d3-AC C16
77	Acylcarnitine	Hydroxyhexadecenoylcarnitine	AC C16:1-OH	d3-AC C16
78	Acylcarnitine	Hexadecadienylcarnitine	AC C16:2	d3-AC C16
79	Acylcarnitine	Hydroxyhexadecadienylcarnitine	AC C16:2-OH	d3-AC C16
80	Acylcarnitine	Hydroxyhexadecanoylcarnitine	AC C16-OH	d3-AC C16
81	Acylcarnitine	Octadecanoylcarnitine	AC C18	d3-AC C16
82	Acylcarnitine	Octadecenoylcarnitine	AC C18:1	d3-AC C16
83	Acylcarnitine	Hydroxyoctadecenoylcarnitine	AC C18:1-OH	d3-AC C16
84	Acylcarnitine	Octadecadienylcarnitine	AC C18:2	d3-AC C16
85	Glycerophospholipid	lysoPhosphatidylcholine acyl C14:0	lysoPC a C14:0	13:0 LPC
86	Glycerophospholipid	lysoPhosphatidylcholine acyl C16:0	lysoPC a C16:0	13:0 LPC
87	Glycerophospholipid	lysoPhosphatidylcholine acyl C16:1	lysoPC a C16:1	17:1 LPC
88	Glycerophospholipid	lysoPhosphatidylcholine acyl C17:0	lysoPC a C17:0	13:0 LPC
89	Glycerophospholipid	lysoPhosphatidylcholine acyl C18:0	lysoPC a C18:0	13:0 LPC
90	Glycerophospholipid	lysoPhosphatidylcholine acyl C18:1	lysoPC a C18:1	17:1 LPC
91	Glycerophospholipid	lysoPhosphatidylcholine acyl C18:2	lysoPC a C18:2	17:1 LPC
92	Glycerophospholipid	lysoPhosphatidylcholine acyl C20:3	lysoPC a C20:3	17:1 LPC

93	Glycerophospholipid	lysoPhosphatidylcholine acyl C20:4	lysoPC a C20:4	17:1 LPC
94	Glycerophospholipid	lysoPhosphatidylcholine acyl C24:0	lysoPC a C24:0	13:0 LPC
95	Glycerophospholipid	lysoPhosphatidylcholine acyl C26:1	lysoPC a C26:1	17:1 LPC
96	Glycerophospholipid	lysoPhosphatidylcholine acyl C28:0	lysoPC a C28:0	13:0 LPC
97	Glycerophospholipid	lysoPhosphatidylcholine acyl C28:1	lysoPC a C28:1	17:1 LPC
98	Glycerophospholipid	Phosphatidylcholine diacyl C24:0	PC aa C24:0	12:0-13:0 PC
99	Glycerophospholipid	Phosphatidylcholine diacyl C26:0	PC aa C26:0	12:0-13:0 PC
100	Glycerophospholipid	Phosphatidylcholine diacyl C28:1	PC aa C28:1	37:4 PC
101	Glycerophospholipid	Phosphatidylcholine diacyl C30:0	PC aa C30:0	12:0-13:0 PC
102	Glycerophospholipid	Phosphatidylcholine diacyl C30:2	PC aa C30:2	37:4 PC
103	Glycerophospholipid	Phosphatidylcholine diacyl C32:0	PC aa C32:0	12:0-13:0 PC
104	Glycerophospholipid	Phosphatidylcholine diacyl C32:1	PC aa C32:1	37:4 PC
105	Glycerophospholipid	Phosphatidylcholine diacyl C32:2	PC aa C32:2	37:4 PC
106	Glycerophospholipid	Phosphatidylcholine diacyl C32:3	PC aa C32:3	37:4 PC
107	Glycerophospholipid	Phosphatidylcholine diacyl C34:1	PC aa C34:1	37:4 PC
108	Glycerophospholipid	Phosphatidylcholine diacyl C34:2	PC aa C34:2	37:4 PC
109	Glycerophospholipid	Phosphatidylcholine diacyl C34:3	PC aa C34:3	37:4 PC
110	Glycerophospholipid	Phosphatidylcholine diacyl C34:4	PC aa C34:4	37:4 PC
111	Glycerophospholipid	Phosphatidylcholine diacyl C36:0	PC aa C36:0	12:0-13:0 PC
112	Glycerophospholipid	Phosphatidylcholine diacyl C36:1	PC aa C36:1	37:4 PC
113	Glycerophospholipid	Phosphatidylcholine diacyl C36:2	PC aa C36:2	37:4 PC
114	Glycerophospholipid	Phosphatidylcholine diacyl C36:3	PC aa C36:3	37:4 PC
115	Glycerophospholipid	Phosphatidylcholine diacyl C36:4	PC aa C36:4	37:4 PC
116	Glycerophospholipid	Phosphatidylcholine diacyl C36:5	PC aa C36:5	37:4 PC
117	Glycerophospholipid	Phosphatidylcholine diacyl C36:6	PC aa C36:6	37:4 PC
118	Glycerophospholipid	Phosphatidylcholine diacyl C38:0	PC aa C38:0	12:0-13:0 PC
119	Glycerophospholipid	Phosphatidylcholine diacyl C38:1	PC aa C38:1	37:4 PC
120	Glycerophospholipid	Phosphatidylcholine diacyl C38:3	PC aa C38:3	37:4 PC
121	Glycerophospholipid	Phosphatidylcholine diacyl C38:4	PC aa C38:4	37:4 PC
122	Glycerophospholipid	Phosphatidylcholine diacyl C38:5	PC aa C38:5	37:4 PC
123	Glycerophospholipid	Phosphatidylcholine diacyl C38:6	PC aa C38:6	37:4 PC
124	Glycerophospholipid	Phosphatidylcholine diacyl C40:1	PC aa C40:1	37:4 PC
125	Glycerophospholipid	Phosphatidylcholine diacyl C40:2	PC aa C40:2	37:4 PC
126	Glycerophospholipid	Phosphatidylcholine diacyl C40:3	PC aa C40:3	37:4 PC
127	Glycerophospholipid	Phosphatidylcholine diacyl C40:4	PC aa C40:4	37:4 PC
128	Glycerophospholipid	Phosphatidylcholine diacyl C40:5	PC aa C40:5	37:4 PC

129	Glycerophospholipid	Phosphatidylcholine diacyl C40:6	PC aa C40:6	37:4 PC
130	Glycerophospholipid	Phosphatidylcholine diacyl C42:0	PC aa C42:0	12:0-13:0 PC
131	Glycerophospholipid	Phosphatidylcholine diacyl C42:1	PC aa C42:1	37:4 PC
132	Glycerophospholipid	Phosphatidylcholine diacyl C42:2	PC aa C42:2	37:4 PC
133	Glycerophospholipid	Phosphatidylcholine diacyl C42:4	PC aa C42:4	37:4 PC
134	Glycerophospholipid	Phosphatidylcholine diacyl C42:5	PC aa C42:5	37:4 PC
135	Glycerophospholipid	Phosphatidylcholine diacyl C42:6	PC aa C42:6	37:4 PC
136	Glycerophospholipid	Phosphatidylcholine diacyl C48:0	PC aa C48:0	12:0-13:0 PC
137	Glycerophospholipid	Phosphatidylcholine acyl-alkyl C30:0	PC ae C30:0	12:0-13:0 PC
138	Glycerophospholipid	Phosphatidylcholine acyl-alkyl C30:1	PC ae C30:1	37:4 PC
139	Glycerophospholipid	Phosphatidylcholine acyl-alkyl C30:2	PC ae C30:2	37:4 PC
140	Glycerophospholipid	Phosphatidylcholine acyl-alkyl C32:1	PC ae C32:1	37:4 PC
141	Glycerophospholipid	Phosphatidylcholine acyl-alkyl C32:2	PC ae C32:2	37:4 PC
142	Glycerophospholipid	Phosphatidylcholine acyl-alkyl C34:0	PC ae C34:0	12:0-13:0 PC
143	Glycerophospholipid	Phosphatidylcholine acyl-alkyl C34:1	PC ae C34:1	37:4 PC
144	Glycerophospholipid	Phosphatidylcholine acyl-alkyl C34:2	PC ae C34:2	37:4 PC
145	Glycerophospholipid	Phosphatidylcholine acyl-alkyl C34:3	PC ae C34:3	37:4 PC
146	Glycerophospholipid	Phosphatidylcholine acyl-alkyl C36:0	PC ae C36:0	12:0-13:0 PC
147	Glycerophospholipid	Phosphatidylcholine acyl-alkyl C36:1	PC ae C36:1	37:4 PC
148	Glycerophospholipid	Phosphatidylcholine acyl-alkyl C36:2	PC ae C36:2	37:4 PC
149	Glycerophospholipid	Phosphatidylcholine acyl-alkyl C36:3	PC ae C36:3	37:4 PC
150	Glycerophospholipid	Phosphatidylcholine acyl-alkyl C36:4	PC ae C36:4	37:4 PC
151	Glycerophospholipid	Phosphatidylcholine acyl-alkyl C36:5	PC ae C36:5	37:4 PC
152	Glycerophospholipid	Phosphatidylcholine acyl-alkyl C38:0	PC ae C38:0	12:0-13:0 PC
153	Glycerophospholipid	Phosphatidylcholine acyl-alkyl C38:1	PC ae C38:1	37:4 PC
154	Glycerophospholipid	Phosphatidylcholine acyl-alkyl C38:2	PC ae C38:2	37:4 PC
155	Glycerophospholipid	Phosphatidylcholine acyl-alkyl C38:3	PC ae C38:3	37:4 PC
156	Glycerophospholipid	Phosphatidylcholine acyl-alkyl C38:5	PC ae C38:5	37:4 PC
157	Glycerophospholipid	Phosphatidylcholine acyl-alkyl C38:6	PC ae C38:6	37:4 PC
158	Glycerophospholipid	Phosphatidylcholine acyl-alkyl C40:1	PC ae C40:1	37:4 PC
159	Glycerophospholipid	Phosphatidylcholine acyl-alkyl C40:2	PC ae C40:2	37:4 PC
160	Glycerophospholipid	Phosphatidylcholine acyl-alkyl C40:3	PC ae C40:3	37:4 PC
161	Glycerophospholipid	Phosphatidylcholine acyl-alkyl C40:4	PC ae C40:4	37:4 PC
162	Glycerophospholipid	Phosphatidylcholine acyl-alkyl C40:5	PC ae C40:5	37:4 PC
163	Glycerophospholipid	Phosphatidylcholine acyl-alkyl C40:6	PC ae C40:6	37:4 PC
164	Glycerophospholipid	Phosphatidylcholine acyl-alkyl C42:0	PC ae C42:0	12:0-13:0 PC

165	Glycerophospholipid	Phosphatidylcholine acyl-alkyl C42:1	PC ae C42:1	37:4 PC
166	Glycerophospholipid	Phosphatidylcholine acyl-alkyl C42:2	PC ae C42:2	37:4 PC
167	Glycerophospholipid	Phosphatidylcholine acyl-alkyl C42:3	PC ae C42:3	37:4 PC
168	Glycerophospholipid	Phosphatidylcholine acyl-alkyl C42:4	PC ae C42:4	37:4 PC
169	Glycerophospholipid	Phosphatidylcholine acyl-alkyl C42:5	PC ae C42:5	37:4 PC
170	Glycerophospholipid	Phosphatidylcholine acyl-alkyl C44:3	PC ae C44:3	37:4 PC
171	Glycerophospholipid	Phosphatidylcholine acyl-alkyl C44:4	PC ae C44:4	37:4 PC
172	Glycerophospholipid	Phosphatidylcholine acyl-alkyl C44:5	PC ae C44:5	37:4 PC
173	Glycerophospholipid	Phosphatidylcholine acyl-alkyl C44:6	PC ae C44:6	37:4 PC
174	Sphingolipid	Hydroxysphingomyeline C14:1	SM (OH) C14:1	SM 12:0
175	Sphingolipid	Sphingomyeline C16:0	SM C16:0	SM 12:0
176	Sphingolipid	Sphingomyeline C16:1	SM C16:1	SM 12:0
177	Sphingolipid	Hydroxysphingomyeline C16:1	SM (OH) C16:1	SM 12:0
178	Sphingolipid	Sphingomyeline C18:0	SM C18:0	SM 12:0
179	Sphingolipid	Sphingomyeline C18:1	SM C18:1	SM 12:0
180	Sphingolipid	Sphingomyeline C20:2	SM C20:2	SM 12:0
181	Sphingolipid	Sphingomyeline C22:3	SM C22:3	SM 12:0
182	Sphingolipid	Hydroxysphingomyeline C22:1	SM (OH) C22:1	SM 12:0
183	Sphingolipid	Hydroxysphingomyeline C22:2	SM (OH) C22:2	SM 12:0
184	Sphingolipid	Sphingomyeline C24:0	SM C24:0	SM 12:0
185	Sphingolipid	Sphingomyeline C24:1	SM C24:1	SM 12:0
186	Sphingolipid	Hydroxysphingomyeline C24:1	SM (OH) C24:1	SM 12:0
187	Sphingolipid	Sphingomyeline C26:0	SM C26:0	SM 12:0
188	Sphingolipid	Sphingomyeline C26:1	SM C26:1	SM 12:0

List 4-Bile Acids

(LC-MS/MS-Analysis)

#	Sub-Class	Name	Abbreviation	Surrogate
189	Non-conjugated bile acid	cholic acid	CA	CA-d5
190	Non-conjugated bile acid	deoxycholic acid	DCA	CDCA-d4
191	Non-conjugated bile acid	chenodeoxycholic acid	CDCA	CDCA-d4
192	Non-conjugated bile acid	ursodeoxycholic acid	UDCA	CDCA-d4
193	Non-conjugated bile acid	lithocholic acid	LCA	LCA-d4
194	Glycine-conjugated bile acid	glycocholic acid	GCA	GCA-d5
195	Glycine-conjugated bile acid	glycodeoxycholic acid	GDCA	GCA-d5
196	Glycine-conjugated bile acid	glycochenodeoxycholic acid	GCDCA	GCA-d5
197	Taurine-conjugated bile acid	taurocholic acid	TCA	TCDCA-d5

198	Taurine-conjugated bile acid	taurodeoxycholic acid	TDCA	TCDCA-d5
199	Taurine-conjugated bile acid	taurochenodeoxycholic acid	TCDCA	TCDCA-d5
200	Taurine-conjugated bile acid	Tauroursodeoxycholic acid	TUDCA	TCDCA-d5
201	Taurine-conjugated bile acid	taurolithocholic acid	TLCA	TCDCA-d5

List 5-Fatty Acids
(LC-MS/MS-Analysis)

#	Sub-Class	Name	Abbreviation	Surrogate
202	Saturated fatty acid	decanoic acid (capric acid)	FA C10:0	d19-C10:0
203	Saturated fatty acid	tetradecanoic acid (myristic acid)	FA C14:0	d27-C14:0
204	Saturated fatty acid	hexadecanoic acid (palmitic acid)	FA C16:0	d31-C16:0
205	polyunsaturated fatty acid	hexadecenoic acid (palmitoleic acid)	FA C16:1	d31-C16:0
206	Saturated fatty acid	octadecanoic acid (stearic acid)	FA C18:0	d35-C18:0
207	polyunsaturated fatty acid	octadecadienoic acid (linoleic acid)	FA C18:2	d35-C18:0
208	polyunsaturated fatty acid	octadecatrienoic acid (γ-linolenic acid)	FA C18:3	d5-C22:6
209	polyunsaturated fatty acid	11, 14-eicosadienoic acid	FA C20:2	d5-C22:6
210	polyunsaturated fatty acid	eicosatrienoic acid (dihomo-γ-linolenic acid)	FA C20:3n6	d5-C22:6
211	polyunsaturated fatty acid	11, 14, 17-eicosatrienoic acid (eicosatrienoic acid)	FA C20:3n3	d5-C22:6
212	polyunsaturated fatty acid	Eicosatetraenoic acid (arachidonic acid)	FA C20:4	d5-C22:6
213	polyunsaturated fatty acid	eicosapentaenoic acid (EPA)	FA C20:5	d5-C20:5
214	polyunsaturated fatty acid	docosatetraenoic acid (adrenic acid)	FA C22:4	d5-C22:6
215	polyunsaturated fatty acid	C22:5 ISOMER 1 (tentatively all-*cis*-4,8,12,15,19-docosapentaenoic acid)[1]	FA C22:5n3c1	d5-C20:5

216	polyunsaturated fatty acid	C22:5 ISOMER 2 (all-*cis*-7,10,13,16,19-docosapentaenoic acid (DPA)	FA C22:5n3c2	d5-C20:5
217	polyunsaturated fatty acid	C22:5 ISOMER 3 (tentatively all-*cis*-4,7,10,13,16-docosapentaenoic acid)[1]	FA C22:5n6c	d5-C20:5
218	polyunsaturated fatty acid	docosahexaenoic acid (DHA)	FA C22:6	d5-C22:6

[1] no authentic standards available, ID of the specific isomers is tentative

Supplementary Table 5. Metabolite Quantification Method Details

Native Analyte	RT (min)	Parent m/z	Product m/z	Internal Standard IS	RT (min)	Parent m/z	Product m/z
Ala	5.7	225.2	44.2	d4-Ala	5.6	229.2	48.2
Arg	4.8	310	44.2	15N2-Arg	4.7	312	219
Asn	5.1	268.2	87	15N2-Asn	5	270.2	89.2
Asp	5.3	269.2	116.2	d3-Asp	5.3	272.2	119.2
Cit	5.2	311.2	113.1	13C-d4-Cit	5.1	316.1	118.2
Gln	5.1	282.2	130	d5-Gln	5	287.2	135
Glu	5.3	283.2	130.2	d3-Glu	5.3	286.2	133.2
Gly	5.4	211.2	75.9	13C2-15N-Gly	5.3	214.2	78.9
His	4.7	291.1	110.2	13C6-His	4.6	297.1	115.2
Ile	6.5	267.3	69	13C6-Ile	6.5	273.2	91.2
Leu	6.5	267.3	43	13C6-Ile	6.5	273.2	91.2
Lys	6.3	417.2	324.2	d6-Orn	6.3	409.2	316.2
Met	6.2	285.1	104.2	d3-Met	6.2	288.1	107.2
Orn	6.3	403.2	310.2	d6-Orn	6.3	409.2	316.2
Phe	6.5	301.2	120.2	d5-Phe	6.5	306.2	125.2
Pro	5.7	251.2	70.3	d7-Pro	5.6	258.2	77.3
Ser	5.2	241.2	60	d3-Ser	5.2	244.2	63
Thr	5.4	255.2	74.1	13C4-Thr	5.5	259.2	77.1
Trp	6.4	340.2	188.2	15N2-Trp	6.4	342.2	189.2
Tyr	5.9	317.2	136.1	d4-Tyr	5.9	321.2	140.1
Val	6.2	253.2	72.2	d8-Val	6.2	261.2	80.2
Ac-Orn	5.3	310.2	217.3	d6-Orn	6.3	409.2	316.2
ADMA	4.9	338.2	46	d7-ADMA	4.9	345.2	77.2
SDMA	5	338.2	307	d7-ADMA	4.9	345.2	77.2
total DMA	4.9	338.2	70.1	d7-ADMA	4.9	345.2	77.2
alpha-AAA	5.4	297.1	144.2	d6-Orn	6.3	409.2	316.2
Carnosine	4.6	362.2	110.1	13C6-His	4.7	297.1	115.2
Creatinine	1	114.1	44.1	d3-Creatinine	1	117.1	47.1

DOPA	5.6	333.1	198.1	d3-DOPA	5.6	336.1	201.1
Dopamine	5.9	289.2	137.2	d4-Dopamine	5.9	293.1	141.1
GABA	5.7	239.2	86.3	d8-Val	6.2	261.2	80.2
Histamine	4.6	247	154	13C6-His	4.6	297.1	115.2
Kynurenine	6.4	344.2	146.2	d4-Tyr	5.8	321.2	140.1
Met-SO	5.1	301.2	88.1	d3-Met	6.1	288.1	107.2
Nitro-Tyr	6.4	362.2	136.1	d4-Tyr	5.8	321.2	140.1
Hyp	5	267.1	68	d7-Pro	5.6	258.2	77.3
PEA	7.1	257.2	105.2	d4-Serotonin	6	316.3	164.2
Putrescine	6.6	266.1	113.9	d4-Putrescine	6.5	363.2	270.1
Sarcosine	5.5	225	90.1	d3-Sarcosine	5.5	228.1	93.1
Serotonin	6.1	312.3	160.2	d4-Serotonin	6.1	316.3	164.2
Spermidine	7	551.2	193.2	d4-Putrescine	6.5	363.2	270.1
Spermine	7.2	743.3	193.2	d4-Putrescine	6.5	363.2	270.1
Taurine	4.8	261	126.1	13C2-Taurine	4.8	263	128
AC C0*	0.8-2.2	162.1	85.1	d9-AC C0	0.8-2.2	171.1	85.1
AC C2*	0.8-2.2	204.1	85.1	d3-AC C2	0.8-2.2	207.1	85.1
AC C3	0.8-2.2	218.1	85.1	d3-AC C3	0.8-2.2	221.1	85.1
AC C3:1	0.8-2.2	216.1	85.1	d3-AC C3	0.8-2.2	221.1	85.1
AC C3-OH	0.8-2.2	234.1	85.1	d3-AC C3	0.8-2.2	221.1	85.1
AC C4	0.8-2.2	232.2	85.1	d3-AC C4	0.8-2.2	235.2	85.1
AC C4:1	0.8-2.2	230.1	85.1	d3-AC C4	0.8-2.2	235.2	85.1
AC C4-OH(C3-DC)	0.8-2.2	248.1	85.1	d3-AC C4	0.8-2.2	235.2	85.1
AC C5	0.8-2.2	246.2	85.1	d9-AC C5	0.8-2.2	255.2	85.1
AC C5:1	0.8-2.2	244.2	85.1	d9-AC C5	0.8-2.2	255.2	85.1
AC C5:1-DC	0.8-2.2	274.1	85.1	d9-AC C5	0.8-2.2	255.2	85.1
AC C5-DC (C6-OH)	0.8-2.2	276.2	85.1	d9-AC C5	0.8-2.2	255.2	85.1
AC C5-M-DC	0.8-2.2	290.2	85.1	d9-AC C5	0.8-2.2	255.2	85.1
AC C5-OH (C3-DC-M)	0.8-2.2	262.2	85.1	d9-AC C5	0.8-2.2	255.2	85.1
AC C6 (C4:1-DC)	0.8-2.2	260.2	85.1	d9-AC C5	0.8-2.2	255.2	85.1
AC C6:1	0.8-2.2	258.2	85.1	d9-AC C5	0.8-2.2	255.2	85.1
AC C7-DC	0.8-2.2	304.2	85.1	d3-AC C8	0.8-2.2	291.2	85.1
AC C8	0.8-2.2	288.2	85.1	d3-AC C8	0.8-2.2	291.2	85.1
AC C9	0.8-2.2	302.2	85.1	d3-AC C8	0.8-2.2	291.2	85.1
AC C10	0.8-2.2	316.2	85.1	d3-AC C8	0.8-2.2	291.2	85.1

AC C10:1	0.8-2.2	314.2	85.1	d3-AC C8	0.8-2.2	291.2	85.1
AC C10:2	0.8-2.2	312.2	85.1	d3-AC C8	0.8-2.2	291.2	85.1
AC C12	0.8-2.2	344.3	85.1	d9-AC C14	0.8-2.2	381.3	85.1
AC C12:1	0.8-2.2	342.3	85.1	d9-AC C14	0.8-2.2	381.3	85.1
AC C12-DC	0.8-2.2	374.3	85.1	d9-AC C14	0.8-2.2	381.3	85.1
AC C14	0.8-2.2	372.3	85.1	d9-AC C14	0.8-2.2	381.3	85.1
AC C14:1	0.8-2.2	370.3	85.1	d9-AC C14	0.8-2.2	381.3	85.1
AC C14:1-OH	0.8-2.2	386.3	85.1	d9-AC C14	0.8-2.2	381.3	85.1
AC C14:2	0.8-2.2	368.3	85.1	d9-AC C14	0.8-2.2	381.3	85.1
AC C14:2-OH	0.8-2.2	384.3	85.1	d9-AC C14	0.8-2.2	381.3	85.1
AC C16	0.8-2.2	400.3	85.1	d3-AC C16	0.8-2.2	403.3	85.1
AC C16:1	0.8-2.2	398.3	85.1	d3-AC C16	0.8-2.2	403.3	85.1
AC C16:1-OH	0.8-2.2	414.3	85.1	d3-AC C16	0.8-2.2	403.3	85.1
AC C16:2	0.8-2.2	396.3	85.1	d3-AC C16	0.8-2.2	403.3	85.1
AC C16:2-OH	0.8-2.2	412.3	85.1	d3-AC C16	0.8-2.2	403.3	85.1
AC C16-OH	0.8-2.2	416.3	85.1	d3-AC C16	0.8-2.2	403.3	85.1
AC C18	0.8-2.2	428.4	85.1	d3-AC C16	0.8-2.2	403.3	85.1
AC C18:1	0.8-2.2	426.4	85.1	d3-AC C16	0.8-2.2	403.3	85.1
AC C18:1-OH	0.8-2.2	442.4	85.1	d3-AC C16	0.8-2.2	403.3	85.1
AC C18:2	0.8-2.2	424.3	85.1	d3-AC C16	0.8-2.2	403.3	85.1
lysoPC a C14:0*	0.8-2.2	468.3	184	13:0 LPC	0.8-2.2	454.3	184
lysoPC a C16:0*	0.8-2.2	496.3	184	13:0 LPC	0.8-2.2	454.3	184
lysoPC a C16:1	0.8-2.2	494.3	184	17:1 LPC	0.8-2.2	508.3	184
lysoPC a C17:0*	0.8-2.2	510.3	184	13:0 LPC	0.8-2.2	454.3	184
lysoPC a C18:0*	0.8-2.2	524.3	184	13:0 LPC	0.8-2.2	454.3	184
lysoPC a C18:1	0.8-2.2	522.3	184	17:1 LPC	0.8-2.2	508.3	184
lysoPC a C18:2	0.8-2.2	520.3	184	17:1 LPC	0.8-2.2	508.3	184
lysoPC a C20:3	0.8-2.2	546.3	184	17:1 LPC	0.8-2.2	508.3	184
lysoPC a C20:4	0.8-2.2	544.3	184	17:1 LPC	0.8-2.2	508.3	184
lysoPC a C24:0*	0.8-2.2	608.4	184	13:0 LPC	0.8-2.2	454.3	184
lysoPC a C26:0*	0.8-2.2	636.5	184	13:0 LPC	0.8-2.2	454.3	184
lysoPC a C26:1	0.8-2.2	634.4	184	17:1 LPC	0.8-2.2	508.3	184
lysoPC a C28:0*	0.8-2.2	664.5	184	13:0 LPC	0.8-2.2	454.3	184
lysoPC a C28:1	0.8-2.2	662.5	184	17:1 LPC	0.8-2.2	508.3	184
PC aa C24:0*	0.8-2.2	622.4	184	12:0-13:0 PC	0.8-2.2	636.5	184
PC aa C26:0	0.8-2.2	650.5	184	12:0-13:0 PC	0.8-2.2	636.5	184

PC aa C28:1	0.8-2.2	676.5	184	37:4 PC	0.8-2.2	796.6	184
PC aa C30:0*	0.8-2.2	706.5	184	12:0-13:0 PC	0.8-2.2	636.5	184
PC aa C30:2	0.8-2.2	702.5	184	37:4 PC	0.8-2.2	796.6	184
PC aa C32:0*	0.8-2.2	734.6	184	12:0-13:0 PC	0.8-2.2	636.5	184
PC aa C32:1	0.8-2.2	732.6	184	37:4 PC	0.8-2.2	796.6	184
PC aa C32:2	0.8-2.2	730.5	184	37:4 PC	0.8-2.2	796.6	184
PC aa C32:3	0.8-2.2	728.5	184	37:4 PC	0.8-2.2	796.6	184
PC aa C34:1	0.8-2.2	760.6	184	37:4 PC	0.8-2.2	796.6	184
PC aa C34:2	0.8-2.2	758.6	184	37:4 PC	0.8-2.2	796.6	184
PC aa C34:3	0.8-2.2	756.6	184	37:4 PC	0.8-2.2	796.6	184
PC aa C34:4	0.8-2.2	754.5	184	37:4 PC	0.8-2.2	796.6	184
PC aa C36:0*	0.8-2.2	790.6	184	12:0-13:0 PC	0.8-2.2	636.5	184
PC aa C36:1	0.8-2.2	788.6	184	37:4 PC	0.8-2.2	796.6	184
PC aa C36:2	0.8-2.2	786.6	184	37:4 PC	0.8-2.2	796.6	184
PC aa C36:3	0.8-2.2	784.6	184	37:4 PC	0.8-2.2	796.6	184
PC aa C36:4	0.8-2.2	782.6	184	37:4 PC	0.8-2.2	796.6	184
PC aa C36:5	0.8-2.2	780.6	184	37:4 PC	0.8-2.2	796.6	184
PC aa C36:6	0.8-2.2	778.5	184	37:4 PC	0.8-2.2	796.6	184
PC aa C38:0*	0.8-2.2	818.7	184	12:0-13:0 PC	0.8-2.2	636.5	184
PC aa C38:1	0.8-2.2	816.7	184	37:4 PC	0.8-2.2	796.6	184
PC aa C38:3	0.8-2.2	812.6	184	37:4 PC	0.8-2.2	796.6	184
PC aa C38:4	0.8-2.2	810.6	184	37:4 PC	0.8-2.2	796.6	184
PC aa C38:5	0.8-2.2	808.6	184	37:4 PC	0.8-2.2	796.6	184
PC aa C38:6	0.8-2.2	806.6	184	37:4 PC	0.8-2.2	796.6	184
PC aa C40:1	0.8-2.2	844.7	184	37:4 PC	0.8-2.2	796.6	184
PC aa C40:2	0.8-2.2	842.7	184	37:4 PC	0.8-2.2	796.6	184
PC aa C40:3	0.8-2.2	840.7	184	37:4 PC	0.8-2.2	796.6	184
PC aa C40:4	0.8-2.2	838.6	184	37:4 PC	0.8-2.2	796.6	184
PC aa C40:5	0.8-2.2	836.6	184	37:4 PC	0.8-2.2	796.6	184
PC aa C40:6	0.8-2.2	834.6	184	37:4 PC	0.8-2.2	796.6	184
PC aa C42:0*	0.8-2.2	874.7	184	12:0-13:0 PC	0.8-2.2	636.5	184
PC aa C42:1	0.8-2.2	872.7	184	37:4 PC	0.8-2.2	796.6	184
PC aa C42:2	0.8-2.2	870.7	184	37:4 PC	0.8-2.2	796.6	184
PC aa C42:4	0.8-2.2	866.7	184	37:4 PC	0.8-2.2	796.6	184
PC aa C42:5	0.8-2.2	864.7	184	37:4 PC	0.8-2.2	796.6	184
PC aa C42:6	0.8-2.2	862.6	184	37:4 PC	0.8-2.2	796.6	184

PC aa C48:0	0.8-2.2	958.8	184	12:0-13:0 PC	0.8-2.2	636.5	184
PC ae C30:0*	0.8-2.2	692.6	184	12:0-13:0 PC	0.8-2.2	636.5	184
PC ae C30:1	0.8-2.2	690.5	184	37:4 PC	0.8-2.2	796.6	184
PC ae C30:2	0.8-2.2	688.5	184	37:4 PC	0.8-2.2	796.6	184
PC ae C32:1	0.8-2.2	718.6	184	37:4 PC	0.8-2.2	796.6	184
PC ae C32:2	0.8-2.2	716.6	184	37:4 PC	0.8-2.2	796.6	184
PC ae C34:0*	0.8-2.2	748.6	184	12:0-13:0 PC	0.8-2.2	636.5	184
PC ae C34:1	0.8-2.2	746.6	184	37:4 PC	0.8-2.2	796.6	184
PC ae C34:2	0.8-2.2	744.6	184	37:4 PC	0.8-2.2	796.6	184
PC ae C34:3	0.8-2.2	742.6	184	37:4 PC	0.8-2.2	796.6	184
PC ae C36:0*	0.8-2.2	776.7	184	12:0-13:0 PC	0.8-2.2	636.5	184
PC ae C36:1	0.8-2.2	774.6	184	37:4 PC	0.8-2.2	796.6	184
PC ae C36:2	0.8-2.2	772.6	184	37:4 PC	0.8-2.2	796.6	184
PC ae C36:3	0.8-2.2	770.6	184	37:4 PC	0.8-2.2	796.6	184
PC ae C36:4	0.8-2.2	768.6	184	37:4 PC	0.8-2.2	796.6	184
PC ae C36:5	0.8-2.2	766.6	184	37:4 PC	0.8-2.2	796.6	184
PC ae C38:0*	0.8-2.2	804.7	184	12:0-13:0 PC	0.8-2.2	636.5	184
PC ae C38:1	0.8-2.2	802.7	184	37:4 PC	0.8-2.2	796.6	184
PC ae C38:2	0.8-2.2	800.7	184	37:4 PC	0.8-2.2	796.6	184
PC ae C38:3	0.8-2.2	798.6	184	37:4 PC	0.8-2.2	796.6	184
PC ae C38:5	0.8-2.2	794.6	184	37:4 PC	0.8-2.2	796.6	184
PC ae C38:6	0.8-2.2	792.6	184	37:4 PC	0.8-2.2	796.6	184
PC ae C40:1	0.8-2.2	830.7	184	37:4 PC	0.8-2.2	796.6	184
PC ae C40:2	0.8-2.2	828.7	184	37:4 PC	0.8-2.2	796.6	184
PC ae C40:3	0.8-2.2	826.7	184	37:4 PC	0.8-2.2	796.6	184
PC ae C40:4	0.8-2.2	824.7	184	37:4 PC	0.8-2.2	796.6	184
PC ae C40:5	0.8-2.2	822.6	184	37:4 PC	0.8-2.2	796.6	184
PC ae C40:6	0.8-2.2	820.6	184	37:4 PC	0.8-2.2	796.6	184
PC ae C42:0*	0.8-2.2	860.8	184	12:0-13:0 PC	0.8-2.2	636.5	184
PC ae C42:1	0.8-2.2	858.7	184	37:4 PC	0.8-2.2	796.6	184
PC ae C42:2	0.8-2.2	856.7	184	37:4 PC	0.8-2.2	796.6	184
PC ae C42:3	0.8-2.2	854.7	184	37:4 PC	0.8-2.2	796.6	184
PC ae C42:4	0.8-2.2	852.7	184	37:4 PC	0.8-2.2	796.6	184
PC ae C42:5	0.8-2.2	850.7	184	37:4 PC	0.8-2.2	796.6	184
PC ae C44:3	0.8-2.2	882.7	184	37:4 PC	0.8-2.2	796.6	184
PC ae C44:4	0.8-2.2	880.7	184	37:4 PC	0.8-2.2	796.6	184

PC ae C44:5	0.8-2.2	878.7	184	37:4 PC	0.8-2.2	796.6	184
PC ae C44:6	0.8-2.2	876.7	184	37:4 PC	0.8-2.2	796.6	184
SM (OH) C14:1	0.8-2.2	689.6	184	SM 12:0	0.8-2.2	647.5	184
SM C16:0	0.8-2.2	703.6	184	SM 12:0	0.8-2.2	647.5	184
SM C16:1	0.8-2.2	701.6	184	SM 12:0	0.8-2.2	647.5	184
SM (OH) C16:1	0.8-2.2	717.6	184	SM 12:0	0.8-2.2	647.5	184
SM C18:0	0.8-2.2	731.6	184	SM 12:0	0.8-2.2	647.5	184
SM C18:1	0.8-2.2	729.6	184	SM 12:0	0.8-2.2	647.5	184
SM C20:2	0.8-2.2	755.6	184	SM 12:0	0.8-2.2	647.5	184
SM C22:3	0.8-2.2	781.6	184	SM 12:0	0.8-2.2	647.5	184
SM (OH) C22:1	0.8-2.2	801.7	184	SM 12:0	0.8-2.2	647.5	184
SM (OH) C22:2	0.8-2.2	799.7	184	SM 12:0	0.8-2.2	647.5	184
SM C24:0	0.8-2.2	815.7	184	SM 12:0	0.8-2.2	647.5	184
SM C24:1	0.8-2.2	813.7	184	SM 12:0	0.8-2.2	647.5	184
SM (OH) C24:1	0.8-2.2	829.7	184	SM 12:0	0.8-2.2	647.5	184
SM C26:0	0.8-2.2	843.7	184	SM 12:0	0.8-2.2	647.5	184
SM C26:1	0.8-2.2	841.7	184	SM 12:0	0.8-2.2	647.5	184
CA	8.8	407.2	407.2 (343.1)	CA-d5	8.7	412.2	412.2
DCA	12.5	391.2	391.2 (343.3)	CDCA-d4	12.2	395.2	395.2
CDCA	12.2	391.2	391.2	CDCA-d4	12.2	395.2	395.2
UDCA	9.3	391.2	391.2	CDCA-d4	12.2	395.2	395.2
LCA	14	375.2	375.2	LCA-d4	14	379.2	379.2
GCA	6.5	464.3	74.0 (402.3)	GCA-d5	6.5	469.3	74
GDCA	9.1	448.3	74.0 (402.2)	GCA-d5	6.5	469.4	74
GCDCA	8.6	448.3	74.0 (386.1)	GCA-d5	6.5	469.4	74
TCA	4.5	514.2	80.0 (107.0)	TCDCA-d5	6.8	503.2	80
TDCA	7.3	498.2	80.0 (124.0)	TCDCA-d5	6.8	503.2	80
TCDCA	6.9	498.2	80.0 (124.0)	TCDCA-d5	6.8	503.2	80
TUDCA	4.3	498.2	80.0 (124.0)	TCDCA-d5	6.8	503.2	80
TLCA	9.4	482.3	80.0 (124.0)	TCDCA-d5	6.8	503.2	80
Glucose	1.1	179	89	13C6 Glucose	1.1	185	92
FA C10:0	13	171.1	171.1	d19-C10:0	12.9	190.1	190.1
FA C14:0	15	227.2	227.2	d27-C14:0	14.9	254.2	254.2
FA C16:0	15.9	255.2	255.2	d31-C16:0	15.7	286.2	286.2

FA C16:1	15.1	253.2	253.2	d31-C16:0	15.7	286.2	286.2
FA C18:0	17	283.2	283.2	d35-C18:0	16.7	318.2	318.2
FA C18:1	16	281.2	281.2 (71.2)	d31-C16:0	15.7	286.2	286.2
FA C18:2	15.3	279.2	279.2	d35-C18:0	16.7	318.2	318.2
FA C18:3	14.8	277.2	59.1	d5-C22:6	14.9	332.1	288.1
FA C20:2	16.1	307.2	307.2	d5-C22:6	14.9	332.1	288.1
FA C20:3n6	15.5	305.2	80	d5-C22:6	14.9	332.1	288.1
FA C20:3n3	15.4	305.2	154.9 (126.7)	d5-C22:6	14.9	332.1	288.1
FA C20:4	15.1	303.2	259.1 (59.0)	d5-C22:6	14.9	332.1	288.1
FA C20:5	14.6	301.2	257.1 (202.9)	d5-C20:5	14.6	306.3	262.1
FA C22:4	15.7	331.2	59	d5-C22:6	14.9	332.1	288.1
FA C22:5n3c1	14.9	329.2	285.2 (58.9)4	d5-C20:5	14.6	306.3	262.1
FA C22:5n3c2	15.1	329.2	285.2 (58.9) 4	d5-C20:5	14.6	306.3	262.1
FA C22:5n6c	15.4	329.2	285.2 (58.9) 4	d5-C20:5	14.6	306.3	262.1
FA C22:6	14.9	327.2	283.1 (229.1)	d5-C22:6	14.9	332.1	288.1

Supplementary Table 6. Median Method Detection Limits for targeted metabolite analysis

**COMPOUND**	**Serum median LOQ (ng/mL)**
Ac-Orn	17.8
ADMA	93.7
Ala	237
alpha-AAA	33
Arg	157
Asn	214
Asp	281
Carnosine	26.7
Cit	142
Creatinine	705
DOPA	79.2
Dopamine	77.8
GABA	206
Gln	655
Glu	197
Gly	248
His	55.2
Histamine	28.4
Ile	90.8
Kynurenine	34.3
Leu	105
Lys	31.75
Met	107
Met-SO	30.4
Nitro-Tyr	42.7
OH-Pro	15.4
Orn	39.1
PEA	4.18
Phe	34.55
Pro	46.6

Putrescine	3.62
Sarcosine	11.5
SDMA	98.8
Ser	91
Serotonin	3.94
Spermidine	27.6
Spermine	76.1
Taurine	51.6
Thr	71.7
total DMA	152
Trp	240
Tyr	113
Val	67.3
CA	28.3
CDCA	25.6
DCA	25.35
GCA	64.7
GCDCA	64.1
GDCA	67.7
LCA	64.15
TCA	64.2
TCDCA	65.15
TDCA	62.6
TLCA	60.75
TUDCA	62.3
UDCA	55.6
Hex	866.5
FA C10:0	6870
FA C14:0	707
FA C16:0	1265
FA C16:1	2030
FA C18:0	802
FA C18:1	1140
FA C18:2	841
FA C18:3	698.5
FA C20:2	394.5

FA C20:3n3	2340
FA C20:3n6	2000
FA C20:4	318
FA C20:5	399.5
FA C22:4	693.5
FA C22:5n3c1	664.5
FA C22:5n3c2	664.5
FA C22:5n6c	665.5
FA C22:6	245
AC C0	214.5
AC C10	14.8
AC C10:1	13.1
AC C10:2	13.1
AC C12	48.8
AC C12:1	48.8
AC C12-DC	48.8
AC C14	48.8
AC C14:1	48.8
AC C14:1-OH	52.75
AC C14:2	48.8
AC C14:2-OH	48.8
AC C16	48.9
AC C16:1	48.9
AC C16:1-OH	48.9
AC C16:2	164
AC C16:2-OH	48.9
AC C16-OH	48.9
AC C18	48.9
AC C18:1	48.9
AC C18:1-OH	46.4
AC C18:2	48.9
AC C2	36.1
AC C3	49.05
AC C3:1	49.05
AC C3-OH	55.4
AC C4	21.4

AC C4:1	21.4
AC C4-OH (C3-DC)	21.4
AC C5	13.5
AC C5:1	22.9
AC C5:1-DC	13.5
AC C5-DC (C6-OH)	13.5
AC C5-M-DC	13.5
AC C5-OH (C3-DC-M)	13.5
AC C6 (C4:1-DC)	13.5
AC C6:1	13.5
AC C7-DC	13.1
AC C8	23.4
AC C9	13.1
lysoPC a C14:0	179
lysoPC a C16:0	235
lysoPC a C16:1	100
lysoPC a C17:0	179
lysoPC a C18:0	235
lysoPC a C18:1	129
lysoPC a C18:2	129
lysoPC a C20:3	217
lysoPC a C20:4	129
lysoPC a C24:0	179
lysoPC a C26:1	100
lysoPC a C28:0	179
lysoPC a C28:1	100
PC aa C24:0	421
PC aa C26:0	421
PC aa C28:1	25.5
PC aa C30:0	522
PC aa C30:2	15.3
PC aa C32:0	522
PC aa C32:1	131
PC aa C32:2	31.1
PC aa C32:3	16.25

PC aa C34:1	512
PC aa C34:2	304
PC aa C34:3	36.2
PC aa C34:4	21.4
PC aa C36:0	475.5
PC aa C36:1	101
PC aa C36:2	162
PC aa C36:3	130
PC aa C36:4	141
PC aa C36:5	307
PC aa C36:6	92.6
PC aa C38:0	522
PC aa C38:1	19.3
PC aa C38:3	50.4
PC aa C38:4	68.8
PC aa C38:5	174
PC aa C38:6	979
PC aa C40:1	19.3
PC aa C40:2	20.5
PC aa C40:3	16.95
PC aa C40:4	19.3
PC aa C40:5	21.9
PC aa C40:6	172
PC aa C42:0	475.5
PC aa C42:1	18.5
PC aa C42:2	18.5
PC aa C42:4	18.5
PC aa C42:5	18.5
PC aa C42:6	26.2
PC aa C48:0	447
PC ae C30:0	475.5
PC ae C30:1	15.7
PC ae C30:2	12.8
PC ae C32:1	26.9
PC ae C32:2	16.25
PC ae C34:0	522

PC ae C34:1	42.6
PC ae C34:2	19.3
PC ae C34:3	18.5
PC ae C36:0	475.5
PC ae C36:1	54.2
PC ae C36:2	28.3
PC ae C36:3	18.5
PC ae C36:4	19.3
PC ae C36:5	32.9
PC ae C38:0	529
PC ae C38:1	31.3
PC ae C38:2	19.3
PC ae C38:3	88.9
PC ae C38:5	34.9
PC ae C38:6	70.5
PC ae C40:1	99.4
PC ae C40:2	33.3
PC ae C40:3	18.5
PC ae C40:4	19.3
PC ae C40:5	23.1
PC ae C40:6	53.5
PC ae C42:0	475.5
PC ae C42:1	19.3
PC ae C42:2	22.9
PC ae C42:3	27.7
PC ae C42:4	31.7
PC ae C42:5	18.5
PC ae C44:3	18.5
PC ae C44:4	19.3
PC ae C44:5	39.3
PC ae C44:6	18.5
SM (OH) C14:1	158
SM (OH) C16:1	158
SM (OH) C22:1	158
SM (OH) C22:2	158
SM (OH) C24:1	132

SM C16:0	170
SM C16:1	132
SM C18:0	170
SM C18:1	139.5
SM C20:2	147
SM C22:3	132
SM C24:0	170
SM C24:1	245
SM C26:0	132
SM C26:1	158
